# Clinical features of anti-mGluR5 encephalitis and comparison according to MRI positivity: a systematic review and analysis

**DOI:** 10.3389/fimmu.2026.1867988

**Published:** 2026-06-05

**Authors:** Jihong Han, Xiaofang Zhong, Lei Liu, Ruxu Zhang, Zhongyang Hu

**Affiliations:** 1 Department of Neurology, The Third Xiangya Hospital, Central South University, Changsha, Hunan, China; 2 Department of Neurology, Xiangxi Tujia and Miao Autonomous Prefecture People’s Hospital, People’s Hospital of Xiangxi Tujia and Miao Autonomous Prefecture, Xiangxi, Hunan, China; 3 Health Management Medical Center, The Third Xiangya Hospital, Central South University, Changsha, Hunan, China

**Keywords:** autoimmune encephalitis, magnetic resonance imaging (MRI), metabotropic glutamate receptor 5 (mGluR5), neuroimmune disease, phenotype spectrum

## Abstract

**Background:**

Anti-metabotropic glutamate receptor 5 (mGluR5) encephalitis is a rare autoimmune neurological disorder with high clinical heterogeneity. The phenotypic spectrum of anti-mGluR5 autoimmune encephalitis (AE) and the relationship between MRI findings and clinical presentations remain to be fully elucidated.

**Methods:**

Previously reported cases were systematically identified through a literature search of PubMed/MEDLINE, Web of Science and Scopus following PRISMA guidelines. Clinical data from eligible cases, including one from our center, were extracted and analyzed. Patients with a predominant encephalitic phenotype were further stratified into MRI-negative and MRI-positive groups, and statistical comparisons were performed.

**Results:**

60 patients were identified, including 40 patients with a predominant AE phenotype. Among the anti-mGluR5 AE patients, common manifestations included cognitive deficits, behavioral and mood disturbances, sleep disturbances, seizures, movement disorders and decreased level of consciousness (dLOC). Tumors occurred in 25.0% of patients, with a higher frequency in Western than Chinese patients (60.0% vs. 4.0%; p <0.001). Brain MRI abnormalities were observed in 52.5% patients, and no significant differences in mRS was observed between MRI-positive and MRI-negative patients. Cerebrospinal fluid (CSF) positivity for anti-mGluR5 antibodies was lower than serum positivity (54.8% vs. 96.8%; p < 0.001). Exploratory analyses suggested cognitive deficits tended to be more frequent in MRI-positive than MRI-negative patients (95.2% vs. 68.4%; p = 0.040). CSF pleocytosis was relatively more common in the MRI-positive group, while sleep disturbances, seizures, dLOC, prodromal symptoms, CSF Oligoclonal bands (OCBs) tended to occur more frequently in the MRI-negative group, though not statistically significant.

**Conclusions:**

The clinical features of anti-mGluR5 AE are heterogeneous. Chinese patients show lower tumor association. Serum antibody testing may be prioritized over CSF testing for diagnosis in suspected anti-mGluR5 patients. MRI abnormalities are observed in approximately half of patients and may not correlate with mRS. Cognitive deficits tend to be more frequent in MRI-positive patients; whereas several other manifestations showed trends, without reaching statistical significance. These observations may suggest hypothesis-generating patterns warranting confirmation.

## 1 Introduction

Glutamate receptors (GluRs) are the main modulators of excitatory synaptic transmission in the central nervous system (CNS) and play essential roles in neuronal communication and network homeostasis (1). GluRs can be classified into ionotropic (iGluRs), such as N-methyl-D-aspartate receptors (NMDARs) and α-amino-3-hydroxy-5-methyl-4-isoxazolepropionic acid receptors (AMPARs), which act as glutamate-gated ion channels, and metabotropic receptors (mGluRs), which couple to G proteins and further activate intracellular signaling (2). mGluRs are classified into three groups (Group I–III) based on sequence homology, pharmacology, and signal transduction mechanisms, with eight cloned genes (mGluR1-mGluR8) identified to date (3). Group I mGluRs, including mGluR1 and mGluR5, which are expressed predominantly on the postsynaptic membrane and are crucial mediators of synaptic transmission, plasticity, neuronal excitability, and adaptive behaviors, thereby further affecting learning and memory processes (4).

Characterized by antibodies targeting the mGluR5, anti-mGluR5 encephalitis was first described as limbic encephalitis associated with Hodgkin lymphoma (Ophelia syndrome) (5). Since the initial description, anti-mGluR5 encephalitis has emerged as a rare but increasingly recognized autoimmune neurological disorder with marked clinical and radiological heterogeneity. Although the disease mainly affects the CNS, recent studies also indicate its potential association with peripheral and autonomic nervous system (6). Clinically, anti-mGluR5 encephalitis presents with a broad spectrum of neurological manifestations, including cognitive deficits, seizures, behavioral and mood disturbances, sleep disorders, movement disorders, as well as less typical symptoms, such as prosopagnosia and hypoesthesia (7). Positive neuroimaging findings, particularly MRI of the limbic structures, are commonly reported; however, extra-limbic regions, including basal ganglia, brainstem, and cerebellum, have also been involved in some patients (8). Notably, a substantial proportion of patients may exhibit normal conventional MRI findings, highlighting the dissociation between clinical severity and structural imaging, which poses diagnostic challenges (7).

Despite increasing reports of individual cases and small series, current evidence remains largely fragmented, and there is a lack of systematic summary of the clinical features of this disease (9). In particular, the relationship between MRI findings and clinical presentations has not been comprehensively evaluated, limiting the ability to develop standardized diagnostic and management strategies. Understanding the spectrum of anti-mGluR5 encephalitis is critical not only for timely diagnosis and treatment, but also for elucidating the underlying pathophysiology of this disease. In this study, we systematically summarize all previously reported cases of anti-mGluR5-associated disorders, including a new case from our institution. By analyzing clinical phenotypes, tumor associations, and auxiliary examinations, including neuroimaging manifestations and serum and CSF antibody positivity rates, we aim to better characterize the clinical features of anti-mGluR5 AE. Our study further explores whether MRI-negative and MRI-positive patients exhibit potential phenotypic differences. These efforts intend to improve understanding of disease heterogeneity and may provide insights into more accurate diagnosis, patient management and future research directions.

## 2 Methods

### 2.1 Literature review and data extraction

**Figure 1:**
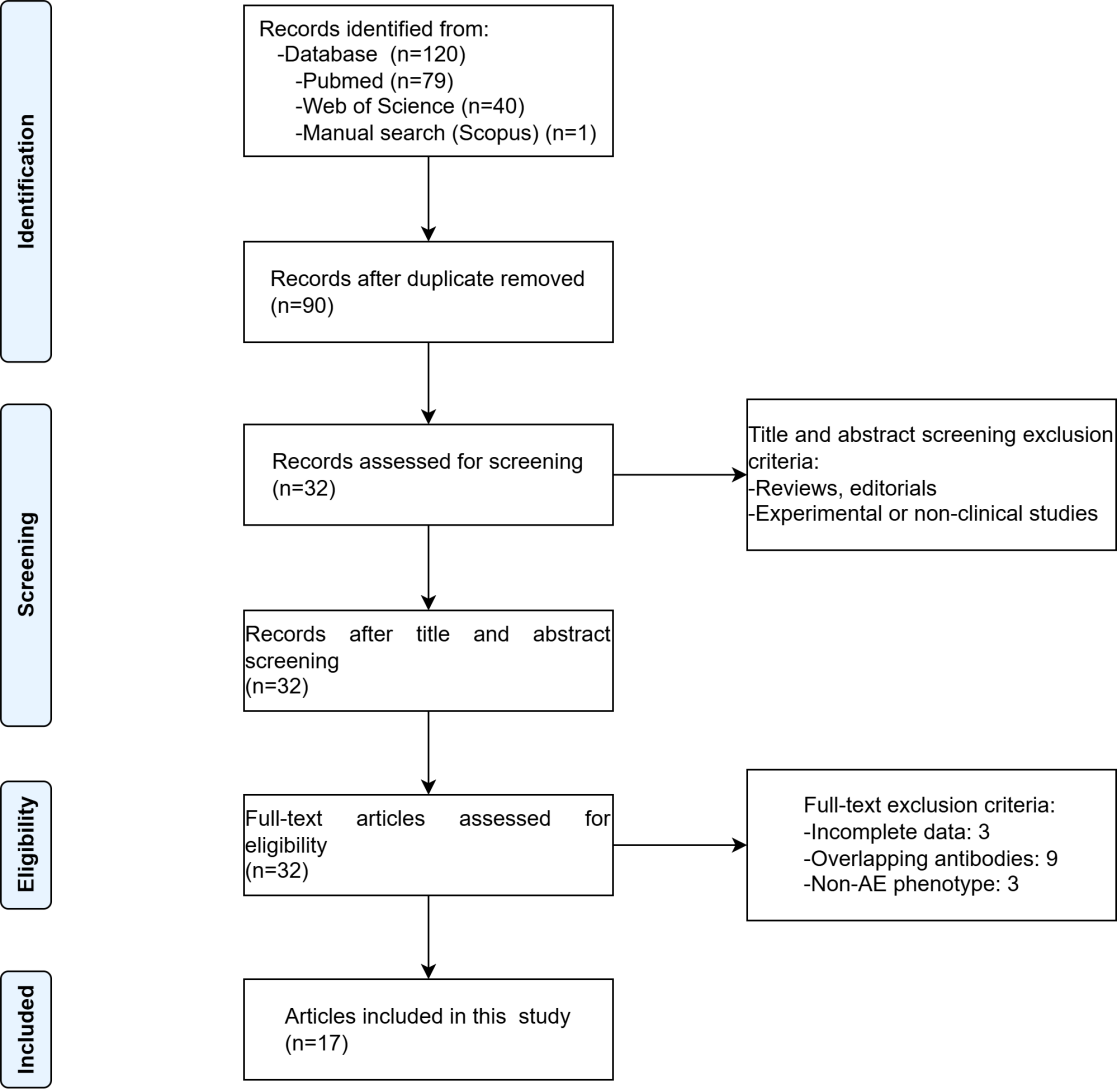
Flow diagram of study selection according to PRISMA guidelines. Records were identified through database searching and screened according to predefined criteria.

Previous cases reported in peer-reviewed journals up to April 2026 were identified through systematic searches of “PubMed/MEDLINE”, “Web of Science” and “Scopus” following the PRISMA guidelines (**Figure 1**). The search strategy included terms such as ‘anti-mGluR5 encephalitis’, ‘anti-mGluR5 antibodies’, and ‘autoimmune’, among others, with no language restrictions applied (**Supplementary Material 1**). In addition to cases identified from the literature, one new case from our institution was included (**Supplementary Material 2**). All cases were confirmed with anti-mGluR5 antibodies by cell-based assays (CBAs) or other validated immunoassays. Eligible patients were systematically reviewed and data on patient demographics, clinical features, auxiliary examinations, tumor associations, treatment regimens, and clinical outcomes were extracted. All information was obtained and further analyzed as reported in the original articles. Data extraction and phenotype classification were independently performed by two investigators using predefined criteria, and discrepancies were resolved through discussion. If consensus could not be achieved, a third investigator was consulted for the final adjudication. To ensure data quality, extracted data were cross-checked against the original publications, including tables, figures, and **Supplementary Materials**.

### 2.2 Inclusion, exclusion and categorization criteria

Patients with confirmed anti-mGluR5 antibodies were included if they presented with a predominant encephalitic phenotype, defined as the presence of at least two core neurological features indicative of central nervous system involvement. These features included cognitive deficits, behavioral and mood disturbances, sleep disturbances, seizures, dLOC and movement disorders (**Table 1**). Patients with non-encephalitic presentations (e.g., Guillain–Barré syndrome, isolated seizures, gastrointestinal symptoms, visual disturbances, or dizziness) or confounding factors, such as overlapping antibodies, infectious or chemical meningoencephalitis, were excluded–from our analyses. For included cases, subgrouping was performed based on the presence or absence of conventional MRI abnormalities detected at any stage of the disease course. Patients were classified as MRI-positive if any conventional brain MRI abnormality was reported, and as MRI-negative if no abnormality was detected throughout follow-up.

**Table 1:** Clinical features of 40 autoimmune encephalitis patients with mGluR5 antibodies.

Variables	Number of patients	Percentage (%)
Demographics		
Median age at onset (IQR)	36 (20,57)	–
Gender, male	22/40	55.0
Clinical features		
Cognitive deficits	33/40	82.5
Memory	25/40	62.5
Disorientation	5/40	12.5
Attention	5/40	12.5
Executive	2/40	5.0
Visuospatial	1/40	2.5
Behavioral and mood disturbances	30/40	75.0
Irritability/mood changes	24/40	60.0
Psychosis/hallucinations	18/40	45.0
Sleep disturbances	19/40	47.5
Seizures	19/40	47.5
Focal onset	8/40	20.0
Generalized onset	8/40	20.0
Status epilepticus	2/40	5.0
Decreased level of consciousness	7/40	17.5
Movement disorders	12/40	30.0
Ataxia	6/40	15.0
Dystonia	3/40	7.5
Tremor	3/40	7.5
Myoclonus	2/40	5.0
Orofacial dyskinesias	1/40	2.5
Other symptoms		
Cranial nerve involvement	5/40	12.5
Meningitis	3/40	7.5
Aphasia	3/40	7.5
Hypoventilation	3/40	7.5
Prosopagnosia	2/40	5.0
Hypoesthesia	2/40	5.0
Prodromal symptoms	24/40	60.0
Headache	9/40	22.5
Weight loss	9/40	22.5
Fever	8/40	20.0
Flu-like symptoms	7/40	17.5
Diarrhea	2/40	5.0
Nausea	2/40	5.0
Erythema/rash	2/40	5.0
Tumor association	10/40	25.0
HD	7/40	17.5
SCLC	1/40	2.5
Acinar adenocarcinoma	1/40	2.5
Gangliocytoma	1/40	2.5
CSF analyses		
CSF pleocytosis	22/40	55.0
CSF OCBs	14/26	53.8
CSF antibody +	17/31	54.8
Autonomic dysfunction	7/40	17.5
Sweating	2/40	5.0
Constipation	2/40	5.0
Fecal incontinence	2/40	5.0
Central hypoventilation	2/40	5.0
Urinary incontinence	1/40	2.5
Urinary retention	1/40	2.5
Median mRS (IQR)		
Peak of the disease	4 (3,4)	–
Last follow-up	1 (0,1)	–
Treatment		
First-line (IVMP, IVIg, PE)	34/39	87.2
Second-line (RTX, MMF)	7/39	17.9
No treatment	4/39	10.3
Follow-up duration (month)	12 (6,20)	–

IQR, interquartile range; HD, Hodgkin lymphoma; SCLC, small cell lung cancer; CSF, cerebrospinal fluid; OCBs, oligoclonal bands; IVMP, intravenous methylprednisolone; IVIg, intravenous immunoglobulin; PE, plasma exchange; RTX, rituximab; MMF, mycophenolate mofetil; +, positivity.

One from our study and 39 from previous studies (7–10, 21, 31–42).

### 2.3 Statistical analyses

Statistical analyses and data visualization were performed using RStudio (version 4.5.3) with the packages: ggplot2, dplyr. Continuous variables were analyzed using the Mann-Whitney U test (Wilcoxon rank-sum test) and results were presented as medians (interquartile range [IQR]). Categorical variables were analyzed using Fisher’s exact test and presented as frequencies and proportions. Paired binary data (such as CSF and serum antibody positivity) was analyzed using the Exact McNemar test. Effect sizes were calculated to provide quantitative comparisons between MRI-positive and MRI-negative groups: risk differences (RDs) for binary outcomes and median differences (estimated using the Hodges-Lehmann method) for continuous or ordinal outcomes. Corresponding 95% confidence intervals (CIs) were reported. All statistical tests were two-sided, with a significance threshold set at p < 0.05. Given the exploratory nature of this study and the small sample size, no adjustments for multiple comparisons were performed, and all analyses were interpreted in an exploratory, hypothesis-generating manner.

## 3 Results

### 3.1 Clinical features of anti-mGluR5 AE

A total of 120 records were identified through database searching. After removal of duplicates, 90 records remained for screening. Following title and abstract screening, 58 articles were excluded, and 32 full-text articles were assessed for eligibility. Of these, 15 records were excluded according to the predefined criteria, leaving 17 studies included in the final analysis (**Figure 1**).

Overall, 60 anti-mGluR5-associated cases with sufficient clinical information were identified, including one case with seizures, dLOC and ataxia from our center (**Supplementary Material 2**). These cases demonstrated a heterogeneous clinical spectrum, encompassing both encephalitic and non-encephalitic presentations. Overlapping antibodies were reported in 13/60 (21.7%) cases and 3/60 (5.0%) cases were complicated by coexisting infections. Tumor associations were observed in 16/60 (26.7%) patients. Detailed information for all cases was provided in **Supplementary Table 1**. Given the clinical heterogeneity and predominance of encephalitic presentations, subsequent analyses focused on patients with a predominant encephalitic phenotype (n=40), as summarized in **Supplementary Table 2**. The clinical information of those excluded from our analyses was presented in **Supplementary Table 3**.

Among these 40 patients, the median age at onset was 36 years (range, 6-75; IQR, 20-57), including 8 (20.0%) children (<18 years of age) and 32 (80.0%) adults. The male/female ratio was 22/18. Prodromal symptoms were reported in 24 (60.0%) patients, including headache (9), weight loss (9), fever (8), flu-like symptoms (7), diarrhea (2), nausea (2), erythema/rash (2). The most frequent neurological manifestations were cognitive deficits (33/40, 82.5%), including impairments in memory, disorientation, attention, visuospatial and executive functions, with memory impairment being the most common subtype (25/40, 62.5%). Behavioral and mood disturbances were observed in 30/40 (75.0%) patients, ranging from irritability or mutism to severe anxiety, depression and full-blown psychosis with abnormal thought processes and hallucinations. Sleep disturbances occurred in 19/40 (47.5%) patients, including somnolence, insomnia and sleep behavior disorders. Seizures were reported in 19/40 (47.5%) patients, including status epilepticus in 2 cases. dLOC was observed in 7/40 (17.5%) patients and movement disorders in 12/40 (30.0%) patients, with ataxia being the most common subtype (6/40, 15.0%). Other less common or atypical presentations included cranial nerve involvement, meningitis, prosopagnosia, hypoventilation, hypoesthesia and aphasia. Autonomic dysfunctions were observed in 7 (17.5%) patients, including sweating, constipation, fecal and urinary incontinence, urinary retention and central hypoventilation. mRS score at disease peak was 4 (IQR, 3-4).

Tumors were identified in 10/40 (25.0%) patients, including Hodgkin lymphoma (n=7), small cell lung cancer (n=1), acinar adenocarcinoma (n=1) and gangliocytoma (n=1). Among reported cases, tumors were more frequently described in Western patients (9/15, 60.0%) than in Chinese patients (1/25, 4.0%) (p < 0.001). Rare cases were noted, including one case following herpes simplex encephalitis (HSE) and one occurring after hematopoietic stem cell transplantation (HSCT). CSF abnormalities were observed in 29/40 (72.5%) patients. Pleocytosis was present in 22 patients (median: 28, range 6–297 white blood cells/mm^3^), and OCBs were detected in 14/26 (53.8%) tested patients. MRI abnormalities were observed in 21/40 (52.5%) patients. Among these, lesions most frequently involved the limbic system (11/21, 52.4%), followed by both the insular cortex (6/21, 28.6%) and extra-limbic cortical or subcortical regions (6/21, 28.6%). Involvement of deep gray matter structures was identified in 3/21 (14.3%) patients, whereas involvement of the brainstem and cerebellum was observed in 4/21 (19.0%). Additional atypical or non-specific imaging findings, such as reversible splenial lesion syndrome (RESLES) and subdural effusion, were reported in 5/21 (23.8%) patients (**Supplementary Table 4**).

Among 31 patients with paired serum and CSF samples, anti-mGluR5 antibodies were detected in both compartments in 16 (51.6%) patients, in serum only in 14 (45.2%) patients, in CSF only in 1 (3.2%) patient. The total positivity rate of CSF antibody was 54.8% and serum was 96.8% among paired samples (p < 0.001). In addition, among patients with single-compartment testing, antibodies were detected in 8 serum-only samples and 1 CSF-only samples. First-line immunotherapy was administered in 34/39 (87.2%) patients, and 7 of them also received second-line immunotherapy. 4/39 (10.3%) patients achieved complete recovery without receiving immunotherapy. Among them, three remained relapse-free at 1 year, and one remained clinically stable over 8 years of follow-up. All patients with associated tumors received chemotherapy or surgical removal as cancer treatment. At the last follow-up (median 12 months), 20/39 (51.3%) patients achieved complete recovery, 19/39 (48.7%) patients had partial recovery. Neurological relapse occurred in 3/39 (7.7%) patients and the median mRS score at follow-up was 1 (IQR, 0-1).

### 3.2 Clinical comparisons between MRI-negative and MRI-positive patients

To explore the association between structural imaging findings and clinical phenotypes, patients with a predominant encephalitic phenotype were categorized into MRI-negative (n=19) and MRI-positive (n=21) groups. Overall, baseline characteristics were broadly comparable between groups, with no statistically significant differences in age at onset, sex distribution, follow-up duration, or treatment strategies (all p > 0.05; **Table 2**).

**Table 2:** Comparison of autoimmune encephalitis with mGluR5 antibodies according to MRI status.

Variables	MRI-negative	MRI-positive	Effect size (95% CI)	P-value
Demographics				
Median age at onset (IQR)	30 (19,45)	46 (29,58)	-10.0 (-25.0 to +5.0)	0.240
Gender, male	9/19 (47.4%)	13/21 (61.9%)	-14.5 (-45.1 to +16.0)	0.525
Clinical features				
Cognitive deficits	13/19 (68.4%)	20/21 (95.2%)	-26.8 (-49.6 to -4.0)	0.040
Behavioral and mood disturbances	15/19 (78.9%)	15/21 (71.4%)	+7.5 (-19.1 to +34.2)	0.721
Sleep disturbances	12/19 (63.2%)	7/21 (33.3%)	+29.8 (+0.2 to +59.4)	0.112
Seizures	11/19 (57.9%)	8/21 (38.1%)	+19.8 (-10.6 to +50.2)	0.342
Decreased level of consciousness	5/19 (26.3%)	2/21 (9.5%)	+16.8 (-6.7 to +40.2)	0.226
Movement disorders	6/19 (31.6%)	6/21 (28.6%)	+3.0 (-25.5 to +31.5)	1.000
Prodromal symptoms	13/19 (68.4%)	11/21 (52.4%)	+16.0 (-13.8 to +45.9)	0.349
Tumor association	4/19 (21.1%)	6/21 (28.6%)	-7.5 (-34.2 to +19.1)	0.721
CSF analyses				
CSF pleocytosis	8/19 (42.1%)	14/21 (66.7%)	-24.6 (-54.6 to +5.4)	0.203
CSF OCBs	10/15 (66.7%)	4/11 (36.4%)	+30.3 (-6.8 to +67.4)	0.233
CSF antibody +	7/14 (50.0%)	10/17 (58.8%)	-8.8 (-43.9 to +26.3)	0.725
Median mRS (IQR)				
Peak of the disease	3 (3,4)	4 (3,4)	0.0 (-1.0 to +1.0)	0.561
Last follow-up	0 (0,1)	1 (0,1)	0.0 (-1.0 to +1.0)	0.368
Follow-up duration	15 (12,19)	12 (5,20)	+6.0 (-0.0 to +11.0)	0.125
Treatment				
First-line (IVMP, IVIg, PE)	17/19 (89.5%)	17/20 (85.0%)	+4.5 (-16.4 to +25.3)	1.000
Second-line (RTX, MMF)	5/19 (26.3%)	2/20 (10.0%)	+16.3 (-7.5 to +40.1)	0.235

Patients with paired serum and CSF samples were included in the analysis of CSF antibody +. Effect size is presented as Hodges-Lehmann median difference for mRS and risk difference (RD) for other clinical features.

MRI, magnetic resonance imaging; CI, confidence interval; CSF, cerebrospinal fluid; OCBs, oligoclonal bands; IQR, interquartile range; IVMP, intravenous methylprednisolone; IVIg, intravenous immunoglobulin; PE, plasma exchange; RTX, rituximab; MMF, mycophenolate mofetil; +, positivity.

**Figure 2:**
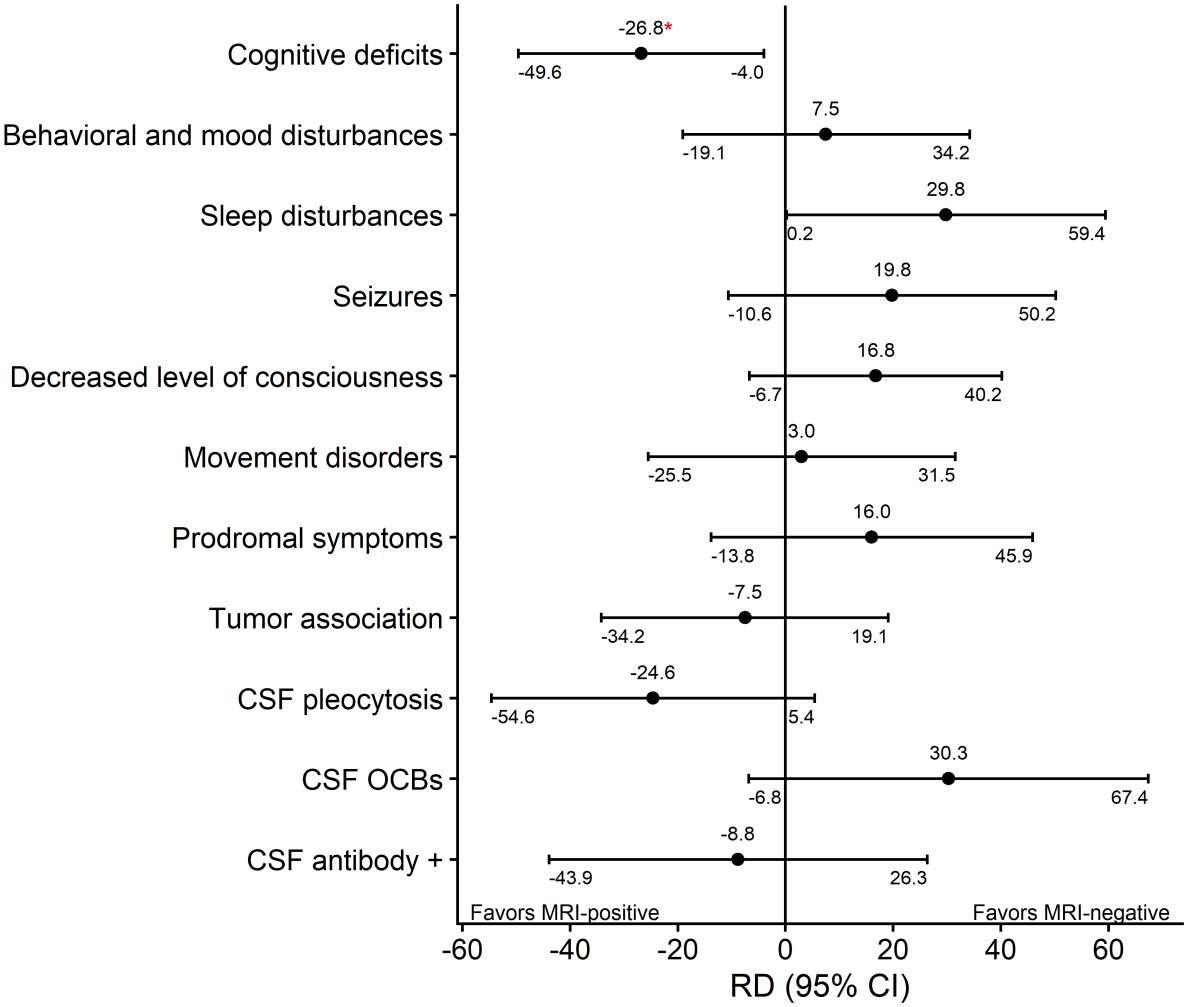
Forest plot of risk differences (RDs) with 95% confidence intervals (CIs) for clinical features between MRI-negative and MRI-positive patients with anti-mGluR5 encephalitis. Negative values indicate a higher frequency in the MRI-positive group, whereas positive values indicate a higher frequency in the MRI-negative group. Statistical significance was indicated by an asterisk for cognitive deficits (p=0.040). Most comparisons did not reach statistical significance, and the results should be interpreted as exploratory. RD, risk difference; CI, confidence interval; CSF, cerebrospinal fluid; OCBs, oligoclonal bands; +, positivity.

Exploratory analyses suggested that cognitive deficits tended to be more frequent in the MRI-positive group (95.2% vs. 68.4%; RD, −26.8%; 95% CI, −49.6 to −4.0; p = 0.040). The frequencies of several other manifestations tended to differ between groups. CSF pleocytosis (66.7% vs. 42.1%; RD, -24.6%; 95% CI, -54.6 to 5.4) was relatively more common in the MRI-positive group than in the MRI-negative group. In contrast, sleep disturbances (63.2% vs. 33.3%; RD, + 29.8%; 95% CI, + 0.2 to 59.4), seizures (57.9% vs. 38.1%; RD, + 19.8%; 95% CI, -10.6 to 50.2), dLOC (26.3% vs. 9.5%; RD, + 16.8%; 95% CI, -6.7 to 40.2), prodromal symptoms (68.4% vs. 52.4%; RD, 16.0%; 95% CI, -13.8 to 45.9) and CSF OCBs (66.7% vs. 36.4%; RD, + 30.3%; 95% CI, -6.8 to 67.4) tended to occur more frequently in the MRI-negative group. Other variables, including behavioral and mood disturbances, movement disorders, tumor association, CSF antibody positivity rate and mRS score at both disease peak and last follow-up, did not demonstrate consistent tendencies (**Figure 2**).

Sensitivity analyses excluding patients with atypical and non-specific MRI findings yielded consistent results with the primary analysis; the effect sizes were generally more pronounced. Cognitive deficits remained more frequent in the MRI-positive group and showed a stronger effect size (RD, -31.6%; 95% CI, -52.5 to -10.1). In addition, CSF pleocytosis demonstrated a strengthened trend toward higher frequency in the MRI-positive group (RD, -28.5%; 95% CI, -59.5 to 2.5) (**Supplementary Table 5**).

## 4 Discussion

This study systematically summarized all previously reported cases, incorporating a novel case from our institution, and provided a comprehensive characterization of the clinical features of anti-mGluR5 AE. We first performed exploratory analyses to assess potential differences in clinical features between MRI-negative and MRI-positive patients. Overall, this study supports the heterogeneous clinical and radiological features of anti-mGluR5 AE and highlights the need for a comprehensive diagnostic approach.

Anti-mGluR5 AE is increasingly recognized as a clinically diverse neurological disorder that extends beyond the traditionally described limbic encephalitis phenotype (9). Our findings support previous observations that cognitive deficits, behavioral and mood disturbances, sleep disturbances, dLOC, seizures, and movement disorders are the most common manifestations, whereas less frequent presentations, including aphasia, hypoventilation, prosopagnosia and hypoesthesia have also been reported (10). The MRI findings similarly reflect this heterogeneity, with involvement not only of limbic structures, but also of extra-limbic cortical and subcortical regions, deep gray matter, brainstem and cerebellum (11, 12). Taken together, these findings support the view that anti-mGluR5 AE is not confined to a classic limbic syndrome. Instead, it should be regarded as a broader autoimmune neuropsychiatric disorder with substantial clinical and radiological heterogeneity.

Tumor association remains an important feature of this disease, and the most common type is Hodgkin lymphoma. As observed in other autoimmune encephalitides, tumors were found more commonly in Western than in Chinese patients (p<0.001) (13). However, this finding should be interpreted with caution, as it may reflect not only possible racial variations in the disease presentation, but also potential reporting bias. In addition, diagnostic technologies, such as positron emission tomography – computed tomography (PET-CT) are more widely available and utilized in western areas, which may also explain the higher tumor association observed in western patients. Some patients may develop tumors during subsequent follow-up, underscoring the necessity for longitudinal surveillance. Notably, 4 patients (10.3%) completely recovered from their illness without specific treatment, and no one relapsed during follow-up. This observation suggests that spontaneous recovery may occur in anti-mGluR5 AE, similar to reports in other forms of AE, including NMDAR encephalitis and anti-leucine-rich glioma-inactivated 1 (LGI1) encephalitis (14, 15). Further studies involving large cohorts with long-term follow-up are needed to determine the prevalence of spontaneous remission in anti-mGluR5 AE.

Cognitive deficits were the only variable that exhibited a statistically significant increase in frequency within the MRI-positive group of anti-mGluR5 AE patients. Other clinical and laboratory features were not statistically significant but showed some interesting directional effect sizes supported by the sensitivity analysis. Specifically, CSF pleocytosis was more common within the MRI-positive group, whereas sleep disturbances, seizures, dLOC, prodromal symptoms, and CSF OCBs showed a higher frequency within the MRI-negative group. The relationship between MRI findings and clinical phenotypes has been explored in other AE, such as NMDAR, LGI1, and anti-gamma-aminobutyric acid type B (GABAB) receptor encephalitis. In these conditions, patients can also exhibit predominantly neuropsychiatric disease with the absence of MRI abnormalities (16–18). No statistical significance in mRS was observed between groups both at disease peak and follow-up. This may indicate that the status of MRI positivity does not necessarily correspond to greater disease severity. Although these observations should be interpreted with caution, they may reflect a tendency for cognitive deficits to occur more frequently in patients with detectable abnormalities on conventional anatomic MRI in anti-mGluR5 AE.

In fact, some patients may be MRI negative throughout their clinical course and manifest with considerable clinical symptoms. mGluR5, widely expressed by interneurons in limbic and frontostriatal circuits, including hippocampus, amygdala and prefrontal cortex, plays a key role in glutamatergic neurotransmitter release, NMDA receptor function and synaptic plasticity (19, 20). Thus, from the perspective of pathophysiology, instead of neuronal loss or demyelination, MRI negativity may reflect a lower degree of injury or dysfunction within distributed neural networks, that cannot be captured by conventional imaging techniques (21, 22). This may be supported by a reported case of mGluR5 AE with immunotherapy-responsive prosopagnosia, in which longitudinal functional MRI showed restoration aligned with clinical recovery (21). The rapid and parallel improvement of both symptoms and functional activation after immunotherapy may be more compatible with reversible antibody-mediated synaptic or network dysfunction than fixed structural damage (7, 23). mGluR5 autoantibodies may act via receptor internalization and disruption of postsynaptic scaffolding proteins (1). They may further lead to impaired synaptic transmission, involving neuronally released glutamate and resulting in altered network excitability (24, 25). Therefore, alternative metabolic or functional imaging techniques, such as fluorodeoxyglucose positron emission tomography (FDG-PET) and task-based Blood Oxygen Level Dependent (BOLD) fMRI, can be more useful for identifying brain changes in these patients (10, 21). In MRI-negative patients, FDG-PET can detect focal or regional metabolic changes that support CNS involvement, whereas task-based BOLD fMRI can indicate network-level functional impairment and recovery, more directly reflecting antibody-mediated synaptic dysfunction (26, 27). These findings underscore that MRI negativity should not preclude the diagnosis of anti-mGluR5 AE. Furthermore, the clinical overlap across MRI-negative and MRI-positive groups in anti-mGluR5 AE can be best described by a continuum of phenotypes rather than distinct sub-groups, consistent with previous conceptualizations of autoimmune encephalitis (28, 29).

Additionally, movement disorders were present in approximately one third of patients, suggesting that such manifestations should receive greater clinical attention in the diagnostic evaluation of anti-mGluR5 AE. The CSF positivity rate for anti-mGluR5 antibodies was lower than the serum positivity rate (54.8% vs. 96.8%; p < 0.001), which contrasts with anti-NMDAR encephalitis, with a CSF positivity rate 100% and a serum positivity rate 85.6% (30). This indicates that in the diagnosis of anti-mGluR5 AE, a negative CSF result should not exclude the disease and serum testing is therefore significant. In clinical practice, it is important to consider other forms of autoimmune encephalitis in the differential diagnosis. Testing both serum and CSF samples with a broad neuronal surface antibody panel may help confirm anti-mGluR5 AE, exclude other autoimmune encephalitides, and identify potential coexisting antibodies. This may therefore provide guidance for subsequent clinical management.

Our index case presented with seizures, dLOC and ataxia while MRI findings were negative. Notably, despite treatment with standardized intravenous immunoglobulin (IVIg) and intravenous methylprednisolone (IVMP) followed by tapering dose of oral steroids, this patient still showed three-fold elevation of serum antibody titers at six-month follow-up without clinical recurrence (**Supplementary Material 2**). This apparent dissociation between antibody levels and clinical manifestations illustrates that, in line with Graus 2016 criteria, clinical assessment should remain the primary basis for diagnosis rather than relying solely on antibody testing (28). Consistent with previous studies of anti-NMDA receptor encephalitis, our single case appears to align with the notion that serum antibody levels may not reliably reflect disease activity (18, 30). However, given this observation is based on a single patient, it should be considered exploratory and require further validation in larger cohorts with long-term follow-up.

On the basis of earlier studies, we systematically summarized all previously reported cases of anti-mGluR5 encephalitis and included a novel case from our center. Focusing on patients with a predominant encephalitic phenotype, we characterized their clinical features and further explored potential phenotypic differences according to MRI status. Cognitive deficits tended to be more frequent in MRI-positive patients. In contrast, several manifestations were more common in MRI-negative patients, but these differences did not reach statistical significance. Due to the small sample size and heterogeneity of data, these findings should be interpreted as hypothesis-generating. Normal MRI is observed in approximately half of anti-mGluR5 patients; thus, the absence of conventional MRI abnormalities does not preclude the diagnosis, particularly when patients present with two or more predominant AE manifestations, including seizures, sleep disturbances, and other symptoms. For patients suspected of anti-mGluR5 AE, paired serum and CSF testing with a broad neuronal surface antibody panel, including mGluR5, is recommended for accurate diagnosis and for the differential diagnosis. Furthermore, functional MRI may help reveal alterations in cerebral functions, which may indirectly reflect neural network dysfunction not captured by conventional MRI, whereas FDG-PET or PET/CT may detect metabolic abnormalities and screen for occult tumors. Collectively, clinicians should integrate clinical manifestations, imaging findings, CSF analysis, tumor screening, and paired antibody testing to facilitate early diagnosis and management.

Noticeably, there are several strengths in our study. First, the application of PRISMA-compliant methodology ensures a systematic and reproducible literature search, enhancing the completeness and transparency of study selection. Second, clear inclusion and exclusion criteria, along with appropriate statistical methods help maintain the focus and reliability of our study. Third, to our knowledge, this is the first study to explore potential clinical differences between MRI-positive and MRI-negative patients with anti-mGluR5 AE. Although the findings require further validation in larger cohorts, they may help refine clinical recognition and generate hypotheses for future research. Finally, by integrating all previously reported cases with a novel case from our institution, this study provides a relatively comprehensive overview of the clinical phenotypic spectrum and heterogeneity of anti-mGluR5 AE.

However, several limitations should be acknowledged. First, as this is a retrospective analysis based on published case reports, there are potential publication biases among the included studies. Second, although all reported cases of this rare disorder are included, the sample size is still relatively small. This limits the statistical power and may lead to wide confidence intervals in our analysis. Third, heterogeneity in diagnostic methods, imaging protocols, antibody testing, and follow-up durations across cases may introduce variability and affect the generalizability of our findings. Finally, as an exploratory analysis, no adjustments for multiple comparisons were performed in this study. Thus, any observed trends should be interpreted cautiously.

Future research should establish larger, multicenter cohorts with standardized and prospective collection of clinical, imaging, and antibody data to improve statistical power and validate our exploratory findings. Advanced functional and metabolic imaging, such as fMRI and FDG-PET, may help clarify mechanisms underlying MRI-negative cases. Longitudinal studies with standardized neurocognitive and antibody assessments are needed to explore predictors of relapse, spontaneous recovery, and long-term functional and cognitive outcomes, potentially informing future diagnostic and management approaches for anti-mGluR5 AE.

In conclusion, we characterized the clinical features of anti-mGluR5 AE, which exhibit significant clinical heterogeneity. A lower tumor association was observed in Chinese patients than in Western patients. Serum antibody testing may be prioritized over CSF testing for diagnosis. MRI abnormalities were detected in approximately half of the patients and did not necessarily correlate with the mRS. Exploratory analysis suggests cognitive deficits tend to be more frequent in MRI-positive patients; whereas several other manifestations show similar trends, without reaching statistical significance. These observations may suggest hypothesis-generating patterns, which warrant confirmation in larger, multicenter cohorts with long-term follow-up.

## Data Availability

The original contributions presented in the study are included in the article/**Supplementary Material**. Further inquiries can be directed to the corresponding author.

## References

[1] Dalmau J Geis C Graus F . Autoantibodies to synaptic receptors and neuronal cell surface proteins in autoimmune diseases of the central nervous system. Physiol Rev. (2017) 97:839–87. doi: 10.1152/physrev.00010.2016. PMID: 28298428 PMC5539405

[2] Faas GC Adwanikar H Gereau RW Saggau P . Modulation of presynaptic calcium transients by metabotropic glutamate receptor activation: a differential role in acute depression of synaptic transmission and long-term depression. J Neurosci. (2002) 22:6885–90. doi: 10.1523/JNEUROSCI.22-16-06885.2002. PMID: 12177186 PMC6757876

[3] De Blasi A Conn PJ Pin J Nicoletti F . Molecular determinants of metabotropic glutamate receptor signaling. Trends Pharmacol Sci. (2001) 22:114–20. doi: 10.1016/s0165-6147(00)01635-7. PMID: 11239574

[4] Scheefhals N MacGillavry HD . Functional organization of postsynaptic glutamate receptors. Mol Cell Neurosci. (2018) 91:82–94. doi: 10.1016/j.mcn.2018.05.002. PMID: 29777761 PMC6276983

[5] Carr I . The Ophelia syndrome: memory loss in Hodgkin’s disease. Lancet. (1982) 1:844–5. doi: 10.1016/s0140-6736(82)91887-6. PMID: 6122069

[6] Yan W Zhao C Zhang H Hu Z Wang C . Case report: Guillain-Barré syndrome characterized by severe headache associated with metabotropic glutamate receptor 5 antibody. Front Immunol. (2022) 13:808131. doi: 10.3389/fimmu.2022.808131. PMID: 35386694 PMC8977415

[7] Spatola M Sabater L Planagumà J Martínez-Hernandez E Armangué T Prüss H . Encephalitis with mGluR5 antibodies: symptoms and antibody effects. Neurology. (2018) 90:e1964–72. doi: 10.1212/WNL.0000000000005614. PMID: 29703767 PMC5980520

[8] Chen S Ren H Lin F Fan S Cao Y Zhao W . Anti-metabotropic glutamate receptor 5 encephalitis: five case reports and literature review. Brain Behav. (2023) 13:e3003. doi: 10.1002/brb3.3003. PMID: 37060179 PMC10175974

[9] Guo K Liu X Gong X Li A Liu Y Li X . Autoimmune encephalitis with mGluR5 antibodies: a case series from China and review of the literature. Front Immunol. (2023) 14:1146536. doi: 10.3389/fimmu.2023.1146536. PMID: 37025999 PMC10070949

[10] Sun Y Tao JX Han X Wang X Zhu Y Lian Y . Clinical features and brain MRI volumetric changes in anti-mGluR5 encephalitis. Ann Clin Transl Neurol. (2023) 10:1407–16. doi: 10.1002/acn3.51831. PMID: 37329164 PMC10424662

[11] Shigemoto R Nomura S Ohishi H Sugihara H Nakanishi S Mizuno N . Immunohistochemical localization of a metabotropic glutamate receptor, mGluR5, in the rat brain. Neurosci Lett. (1993) 163:53–7. doi: 10.1016/0304-3940(93)90227-c. PMID: 8295733

[12] Romano C Sesma MA McDonald CT O’Malley K Van den Pol AN Olney JW . Distribution of metabotropic glutamate receptor mGluR5 immunoreactivity in rat brain. J Comp Neurol. (1995) 355:455–69. doi: 10.1002/cne.903550310. PMID: 7636025

[13] Shan W Yang H Wang Q . Neuronal surface antibody-medicated autoimmune encephalitis (limbic encephalitis) in China: a multiple-center, retrospective study. Front Immunol. (2021) 12:621599. doi: 10.3389/fimmu.2021.621599. PMID: 33679765 PMC7928315

[14] Evoli A Spinelli P Frisullo G Alboini PE Servidei S Marra C . Spontaneous recovery from anti-NMDAR encephalitis. J Neurol. (2012) 259:1964–6. doi: 10.1007/s00415-012-6457-y. PMID: 22392580

[15] Pollak TA Moran N . Emergence of new-onset psychotic disorder following recovery from LGI1 antibody-associated limbic encephalitis. BMJ Case Rep. (2017) 2017:bcr2016218328. doi: 10.1136/bcr-2016-218328. PMID: 28363946 PMC5388006

[16] van Sonderen A Thijs RD Coenders EC Jiskoot LC Sanchez E de Bruijn MAAM . Anti-LGI1 encephalitis: clinical syndrome and long-term follow-up. Neurology. (2016) 87:1449–56. doi: 10.1212/WNL.0000000000003173. PMID: 27590293

[17] Lancaster E Lai M Peng X Hughes E Constantinescu R Raizer J . Antibodies to the GABA(B) receptor in limbic encephalitis with seizures: case series and characterisation of the antigen. Lancet Neurol. (2010) 9:67–76. doi: 10.1016/S1474-4422(09)70324-2. PMID: 19962348 PMC2824142

[18] Gong X Chen C Liu X Lin J Li A Guo K . Long-term functional outcomes and relapse of anti-NMDA receptor encephalitis: a cohort study in Western China. Neurol Neuroimmunol Neuroinflamm. (2021) 8:e958. doi: 10.1212/NXI.0000000000000958. PMID: 33589542 PMC8105891

[19] Niswender CM Conn PJ . Metabotropic glutamate receptors: physiology, pharmacology, and disease. Annu Rev Pharmacol Toxicol. (2010) 50:295–322. doi: 10.1146/annurev.pharmtox.011008.145533. PMID: 20055706 PMC2904507

[20] Conn PJ Pin JP . Pharmacology and functions of metabotropic glutamate receptors. Annu Rev Pharmacol Toxicol. (1997) 37:205–37. doi: 10.1146/annurev.pharmtox.37.1.205. PMID: 9131252

[21] Prüss H Rothkirch M Kopp U Hamer HM Hagge M Sterzer P . Limbic encephalitis with mGluR5 antibodies and immunotherapy-responsive prosopagnosia. Neurology. (2014) 83:1384–6. doi: 10.1212/WNL.0000000000000865. PMID: 25194012

[22] Probasco JC Solnes L Nalluri A Cohen J Jones KM Zan E . Decreased occipital lobe metabolism by FDG-PET/CT: an anti-NMDA receptor encephalitis biomarker. Neurol Neuroimmunol Neuroinflamm. (2018) 5:e413. doi: 10.1212/NXI.0000000000000413. PMID: 29159205 PMC5688263

[23] Moreno-Ajona D Prieto E Grisanti F Esparragosa I Sánchez Orduz L Gállego Pérez-Larraya J . 18F-FDG-PET imaging patterns in autoimmune encephalitis: impact of image analysis on the results. Diagnost (Basel). (2020) 10:356. doi: 10.3390/diagnostics10060356. PMID: 32486044 PMC7344773

[24] Mitoma H Honnorat J Yamaguchi K Manto M . Fundamental mechanisms of autoantibody-induced impairments on ion channels and synapses in immune-mediated cerebellar ataxias. Int J Mol Sci. (2020) 21:4936. doi: 10.3390/ijms21144936. PMID: 32668612 PMC7404345

[25] Barone A Vellucci L Nasti A Mazza B Iannotta F Iasevoli F . Glutamate metabotropic receptors-linked postsynaptic density proteins: an emergent hub for antipsychotics’ regulation of synaptic plasticity and metaplasticity. Biomolecules. (2026) 16:324. doi: 10.3390/biom16020324. PMID: 41750392 PMC12938303

[26] Wang J Guo K Cui B Hou Y Zhao G Lu J . Individual [18F]FDG PET and functional MRI based on simultaneous PET/MRI may predict seizure recurrence after temporal lobe epilepsy surgery. Eur Radiol. (2022) 32:3880–8. doi: 10.1007/s00330-021-08490-9. PMID: 35024947

[27] Duncan JS Winston GP Koepp MJ Ourselin S . Brain imaging in the assessment for epilepsy surgery. Lancet Neurol. (2016) 15:420–33. doi: 10.1016/S1474-4422(15)00383-X. PMID: 26925532 PMC6736670

[28] Graus F Titulaer MJ Balu R Benseler S Bien CG Cellucci T . A clinical approach to diagnosis of autoimmune encephalitis. Lancet Neurol. (2016) 15:391–404. doi: 10.1016/S1474-4422(15)00401-9. PMID: 26906964 PMC5066574

[29] Abboud H Probasco JC Irani S Ances B Benavides DR Bradshaw M . Autoimmune encephalitis: proposed best practice recommendations for diagnosis and acute management. J Neurol Neurosurg Psychiatry. (2021) 92:757–68. doi: 10.1136/jnnp-2020-325300. PMID: 33649022 PMC8223680

[30] Gresa-Arribas N Titulaer MJ Torrents A Aguilar E McCracken L Leypoldt F . Antibody titres at diagnosis and during follow-up of anti-NMDA receptor encephalitis: a retrospective study. Lancet Neurol. (2014) 13:167–75. doi: 10.1016/S1474-4422(13)70282-5. PMID: 24360484 PMC4006368

[31] Chen J Tang X Wang L Guo D Cao L Lu K . Spontaneous partial resolution of autoimmune-mediated brain MRI abnormalities before immunotherapy in anti-metabotropic glutamate receptor 5 encephalitis: a case report. Front Immunol. (2025) 16:1568005. doi: 10.3389/fimmu.2025.1568005. PMID: 40519912 PMC12162606

[32] Hansen N Rentzsch K Hirschel S Wiltfang J Schott BH Malchow B . Persisting verbal memory encoding and recall deficiency after mGluR5 autoantibody-mediated encephalitis. Brain Sci. (2023) 13:1537. doi: 10.3390/brainsci13111537. PMID: 38002497 PMC10669453

[33] Mat A Adler H Merwick A Chadwick G Gullo G Dalmau JO . Ophelia syndrome with metabotropic glutamate receptor 5 antibodies in CSF. Neurology. (2013) 80:1349–50. doi: 10.1212/WNL.0b013e31828ab325. PMID: 23486886 PMC3656459

[34] Pa S O T-G P L-I P C-C . mGlur5 encephalitis causing myoclonus-ataxia syndrome and psychosis: a case report. Movem Disord Clin Pract. (2024) 11:S26–S29. doi: 10.1002/mdc3.14136. PMID: 38923251 PMC11322582

[35] Zhang Y Lian B Yang S Huang X Zhou Y Cao L . Metabotropic glutamate receptor 5-related autoimmune encephalitis with reversible splenial lesion syndrome following SARS-CoV-2 vaccination. Med (Baltimore). (2023) 102:e32971. doi: 10.1097/MD.0000000000032971. PMID: 36800591 PMC9936002

[36] Pedrosa DA Ferreira JHF Gleizer R Carra RB de Carvalho RM Endmayr V . Encephalitis associated with anti-mGluR5 antibodies. Pract Neurol. (2024) 24:306–9. doi: 10.1136/pn-2024-004089. PMID: 38423754

[37] Guevara C Farias G Silva-Rosas C Alarcon P Abudinen G Espinoza J . Encephalitis associated to metabotropic glutamate receptor 5 (mGluR5) antibodies in cerebrospinal fluid. Front Immunol. (2018) 9:2568. doi: 10.3389/fimmu.2018.02568. PMID: 30455705 PMC6230718

[38] Niu Z Chen S Wang J Yu M Ren J Liu R . Clinical spectrum and outcomes of anti-metabotropic glutamate receptor 5 encephalitis in Chinese patients: a case report and literature review. Front Immunol. (2025) 16:1656832. doi: 10.3389/fimmu.2025.1656832. PMID: 41280886 PMC12631469

[39] Yang X Liu Q Lai M-F Ma X-H Hao X-T Xu J-J . Case report: Orthostatic leg tremor as the initial manifestation in a patient with metabotropic glutamate receptor-5 encephalitis without cortical dysfunction: complexities in identification and treatment. Front Neurol. (2023) 14:1288075. doi: 10.3389/fneur.2023.1288075. PMID: 38162450 PMC10755007

[40] Zhang M-M Wang J Sun D Wang J-X Zhang J-H Xu J-W . Case report: Autoimmune encephalitis and other neurological syndromes with rare neuronal surface antibody in children after hematopoietic stem cell transplantation. Front Immunol. (2023) 14:1274420. doi: 10.3389/fimmu.2023.1274420. PMID: 37954605 PMC10637573

[41] Lancaster E Martinez-Hernandez E Titulaer MJ Boulos M Weaver S Antoine J-C . Antibodies to metabotropic glutamate receptor 5 in the Ophelia syndrome. Neurology. (2011) 77:1698–701. doi: 10.1212/WNL.0b013e3182364a44. PMID: 22013185 PMC3208954

[42] Shi K Zhao H Li Y Li X Chen W . Anti-metabolic glutamate receptor 5 encephalitis with gangliocytoma: a case and review of the literature. BMC Neurol. (2024) 24:27. doi: 10.1186/s12883-024-03528-z. PMID: 38218780 PMC10787404

