# Supplementary material for: Clinical features of anti-mGluR5 encephalitis and comparison according to MRI positivity: a systematic review and analysis
Source: Front Immunol. 2026 Jun 5;17:1867988. doi: 10.3389/fimmu.2026.1867988 (PMC13254280; doi:10.3389/fimmu.2026.1867988)
Supplement: Supplementary file 1 [file SupplementaryFile1.docx]

Supplementary Material 1

1. Pubmed:

( "Metabotropic Glutamate Receptor 5"[Mesh] OR mGluR5[Title/Abstract] OR mGlu5[Title/Abstract] OR "metabotropic glutamate receptor 5"[Title/Abstract] OR GRM5[Title/Abstract] ) AND ( Humans[Mesh] ) NOT ( animals[Mesh] NOT humans[Mesh] ) AND ( encephalitis[Title/Abstract] OR "autoimmune encephalitis"[Title/Abstract] OR "limbic encephalitis"[Title/Abstract] OR antibody[Title/Abstract] OR antibodies[Title/Abstract] OR autoantibody[Title/Abstract] OR autoantibodies[Title/Abstract] OR "anti-mGluR5"[Title/Abstract] OR "mGluR5 encephalitis"[Title/Abstract] OR "Ophelia syndrome"[Title/Abstract] )

1. Web of Science

("mGluR5" OR "metabotropic glutamate receptor 5") AND ("anti-mGluR5" OR "mGluR5 antibody" OR "mGluR5 antibodies") AND (autoimmune OR immun* OR paraneoplastic OR neurolog*)

1. Scopus

( ( "mGluR5" OR "metabotropic glutamate receptor 5" ) AND ( "anti-mGluR5" OR "mGluR5 antibody" OR "mGluR5 antibodies" OR autoantibody* ) )
