# Supplementary material for: Clinical features of anti-mGluR5 encephalitis and comparison according to MRI positivity: a systematic review and analysis
Source: Front Immunol. 2026 Jun 5;17:1867988. doi: 10.3389/fimmu.2026.1867988 (PMC13254280; doi:10.3389/fimmu.2026.1867988)
Supplement: Supplementary file 2 [file SupplementaryFile2.docx]

Supplementary Material 2

An 18-year-old female presented with sudden transient loss of consciousness after standing up, accompanied by brief limbs convulsions. Soon, her consciousness turned back with no other discomforts. Two days later, the same symptoms happened again and after regaining awareness, she developed generalized immobility with reduced responsiveness of her bodies and showed a decreased level of consciousness, accompanied by asthenia, floppiness, limb numbness, dyspnea, chest tightness, nausea, diarrhea and headache. The patient, a student by profession, had no significant medical or family history but experienced a 15 kg weight loss over the past 3 months. Upon admission to the local hospital, she complained of headache, predominantly on the left side, with left-sided dental pain and percussion tenderness in the upper left molars. The pain was alleviated after ibuprofen administration but other symptoms didn’t improve after being treated with normal saline and potassium. Physical examination showed no abnormality but neurological examination revealed limitation of eye abduction with about 4 mm of scleral show. Muscle strength (5/5) and tone were normal in all limbs. Bilateral ankle clonus and Hoffman signs were positive. Coordination testing showed normal finger-nose test and heel-to-shin maneuvers, but Romberg sign was positive with instability both with eyes open and closed, and her gait was affected significantly. The Scale for the Assessment and Rating of Ataxia (SARA) scored 12 (gait 6, stance 6) and the modified Rankin Scale (mRS) score was 4 when admission. Sensory examination showed decreased deep and superficial sensation in the fingers of both hands and below the knees. No typical epileptic manifestations were observed after admission. Considering these signs, the lesion was first located to the cervical spinal cord. But non-contrast MRI of the cervical spine showed no significant abnormalities explainable for this condition and the electrodiagnostic studies were unremarkable. A further brain MRI with DWI and MRA were also negative **(Figure 1)**. Laboratory tests revealed a decreased total protein count of 55.1 g/L, globulin of 18.4 g/L and prealbumin of 140.3 mg/L. Lumber puncture showed a normal opening pressure of 180 mmH_2_0, and cerebrospinal fluid (CSF) analysis, including routine analysis, biochemical analysis, cytology and bacterial culture, was all within normal limits. Echocardiography and a 2-hour video electroencephalogram (EEG) revealed no abnormalities. Comprehensive color Doppler ultrasonography of the abdomen, pelvis, and urinary system was negative, which excluded the possibility of tumors.

To confirm the diagnosis and optimize subsequent treatment, one week after disease onset, the patient was referred to our tertiary medical center. A thorough laboratory tests, including complete blood count (CBC), C-reactive protein (CRP), magnesium, phosphorus, liver and renal function tests, serum electrolytes, autoimmune panel, ANA profile, ANCA-associated vasculitis panel, anticardiolipin antibodies (ACA), arterial blood gas (ABG) analysis were conducted and all received negative results except for hypoproteinemia, which excluded the possibility of metabolic encephalopathies. The serological testing for autoimmune peripheral neuropathy here in our hospital was negative. Then the lumber puncture was performed again with a normal opening pressure of 150 mmH_2_0, but the patient experienced persistent pain at the lumber puncture site for the next following 2 days, which was poorly relieved by rotundine and ibuprofen but responded well to bucinnazine. The CSF analysis was within normal limits. Serum and CSF neurological examinations identified positive OCBs (oligoclonal bands) in CSF, classified as Pattern 2. Detected with commercial cell-based assays (CBAs; Euroimmun, Lübeck, Germany), serum anti-mGluR5 antibodies were identified as positive (1:32) **(Figure 2)**, confirming the diagnosis of autoimmune encephalitis, although the antibodies were not identified in the CSF. All other tested antibodies related to encephalitis and epilepsy were tested negative, including those against N-methyl-D-aspartate receptor (NMDAR), α-amino-3-hydroxy-5-methyl-4-isoxazolepropionic acid receptor 1 (AMPAR1), α-amino-3-hydroxy-5-methyl-4-isoxazolepropionic acid receptor 2 (AMPAR2), Gamma-aminobutyric acid type B receptor (GABA B receptor), leucine-rich glioma-inactivated protein 1 (LGI1), contactin-associated protein-like 2 (CASPR2), dipeptidyl-peptidase-like protein 6 (DPPX), IgLON family member 5 (IgLON5), glial fibrillary acidic protein-α (GFAP-α), glial fibrillary acidic protein-ε (GFAP-ε), Hu, paraneoplastic Ma antigen 2 (PNMA2), collapsiin response mediator protein 5 (CRMP5), amphiphysin. Psychological assessment revealed mild depressive symptoms with the Self-Rating Depression Scale (SDS) scoring 56, and clinically significant anxiety with the Self-Rating Anxiety Scale (SAS) scoring 43. A 16-hour multimodal video-EEG monitoring was performed and revealed spike-and-wave and polyspike-and-wave complexes (interictal epileptiform discharges) predominantly in the right frontotemporal regions, accompanied by diffuse slow waves **(Figure 2)**. Once the diagnosis was established, three weeks after the disease onset, the patient received a 5-day course of intravenous immunoglobulin (IVIg, 0.4 g/kg/day) together with oral prednisone (60 mg/day, tapered by 5 mg every 2 weeks) for the treatment of encephalitis, while lacosamide was initiated as antiseizure therapy. Interestingly, from the onset of disease to the day of treatment, the patient’s symptoms improved gradually. In our hospital, no symptoms of epileptic seizures were observed, and the ataxia progressively ameliorated. At the initiation of immunotherapy, the SARA was 6(gait 3, stance 3) and mRS score was 3. After the treatment, the mRS score decreased to 2.

At 6-month of follow-up, the family reported that no seizure manifestations had been observed and a subsequent 30-minute EEG screening showed no epileptiform abnormalities. Brain MRI remained negative **(Figure 1)**. Compared with the findings at admission, the ataxia improved markedly, but she still presented with instability during tandem gait, positive Romberg sign both with eyes open and closed. Besides, she complained of paroxysmal pain in both calves and heels, occasionally accompanied by painful spasms of the calves. The SARA score was 2 (gait 1, stance 1) and the mRS score was 1. A following CBA of anti-mGluR5 antibody revealed that the serum titers rebounded to 1:100 **(Figure 1)**. To minimize the risk of relapse, maintenance therapy with oral prednisone (10 mg/day) was continued and mycophenolate mofetil (MMF) was initiated at 0.25 g twice daily and titrated to 0.75 g twice daily.

At 12-month of follow-up, the patient continued oral prednisone at the same dosage and MMF at 0.75 g twice daily, and remained functionally independent and exhibited no evidence of clinical relapse with the mRS scoring 0.

Figure 1. Longitudinal findings of the patient. (A-B). Normal FLAIR signals in the cerebral and limbic regions at baseline. (C-D). Normal FLAIR signals in the cerebral and limbic regions at 6 months follow-up.


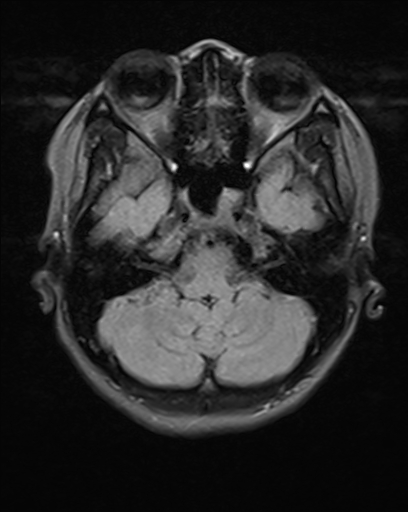

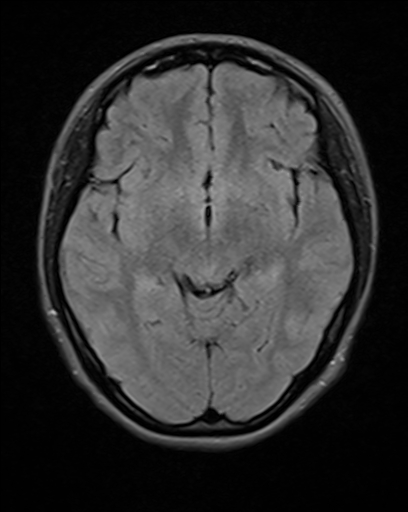

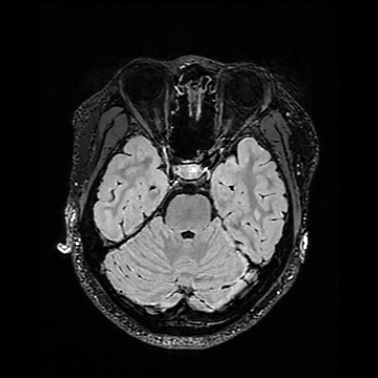

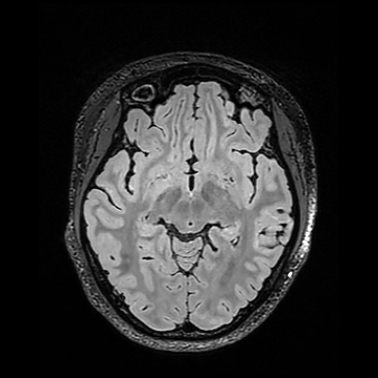


**A**

**B**

**C**

**D**

Figure 2. EEG and serum antibodies of the patient. (A) EEG revealed spike-and-wave and polyspike-and-wave complexes, accompanied by diffuse slow waves. (B) The detection of serum mGluR5 antibodies with CBA at baseline showed a titer of 1:32. (C) Serum mGluR5 antibodies at 6-month follow-up showed a titer of 1:100.


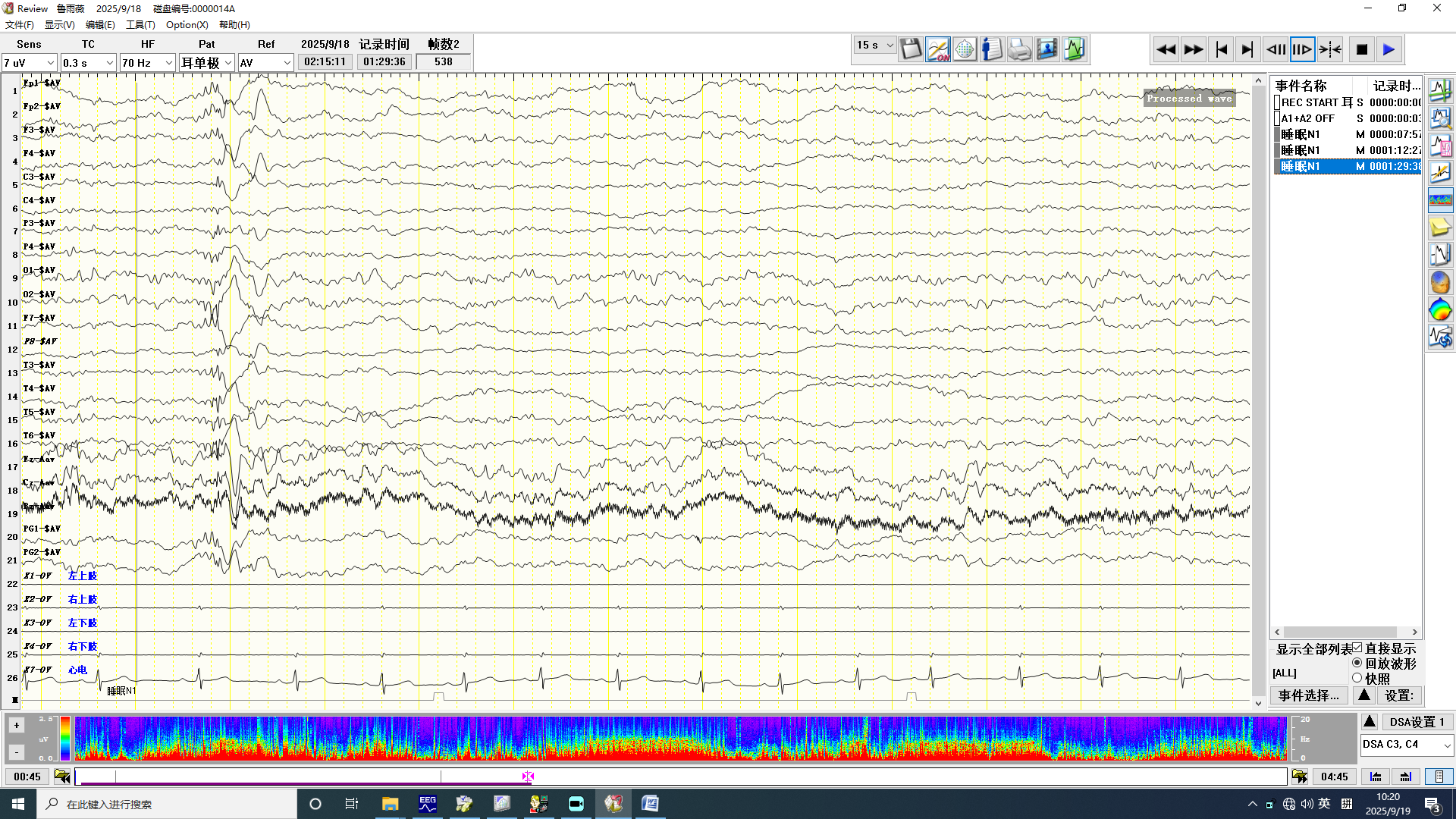

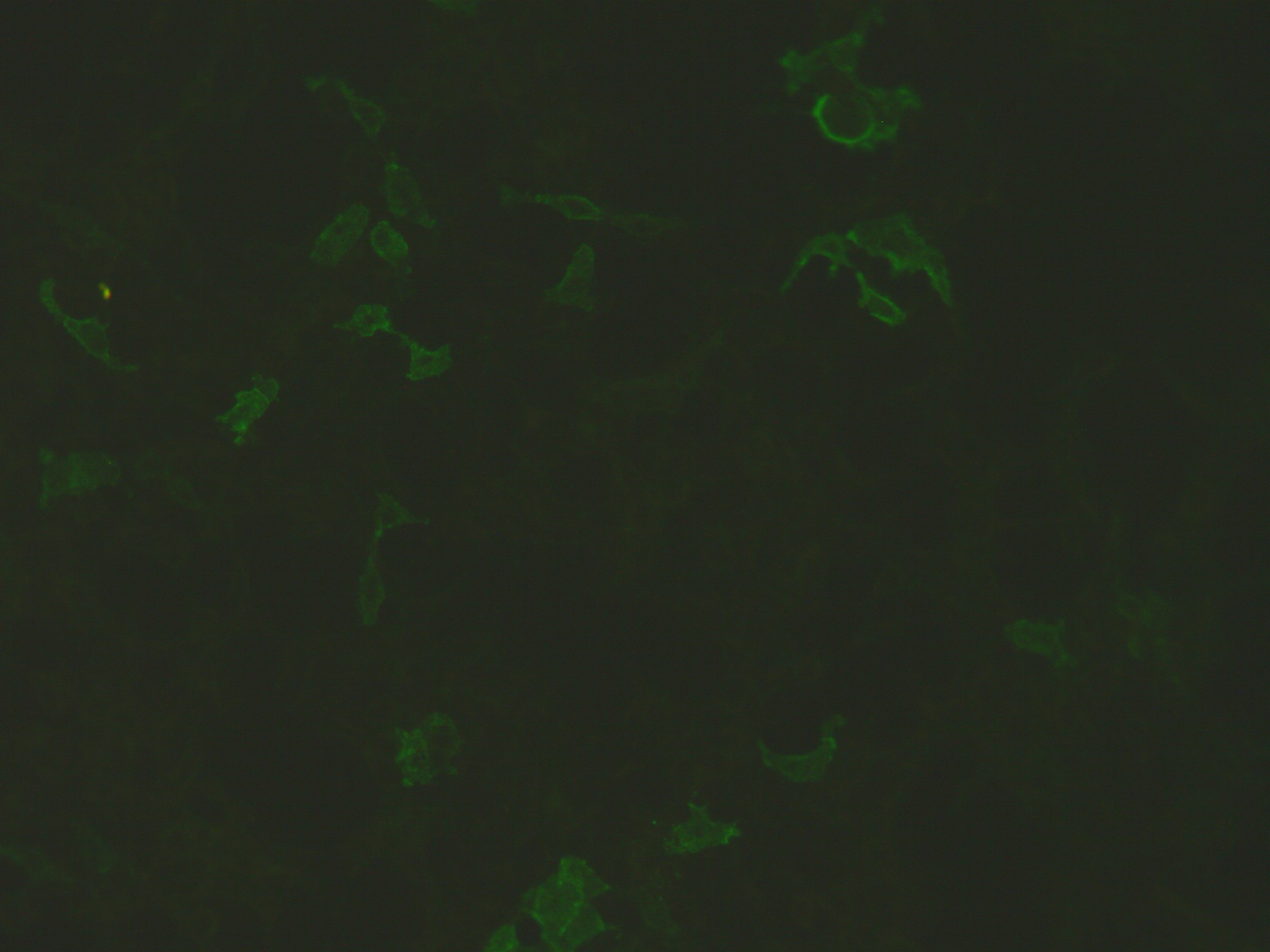

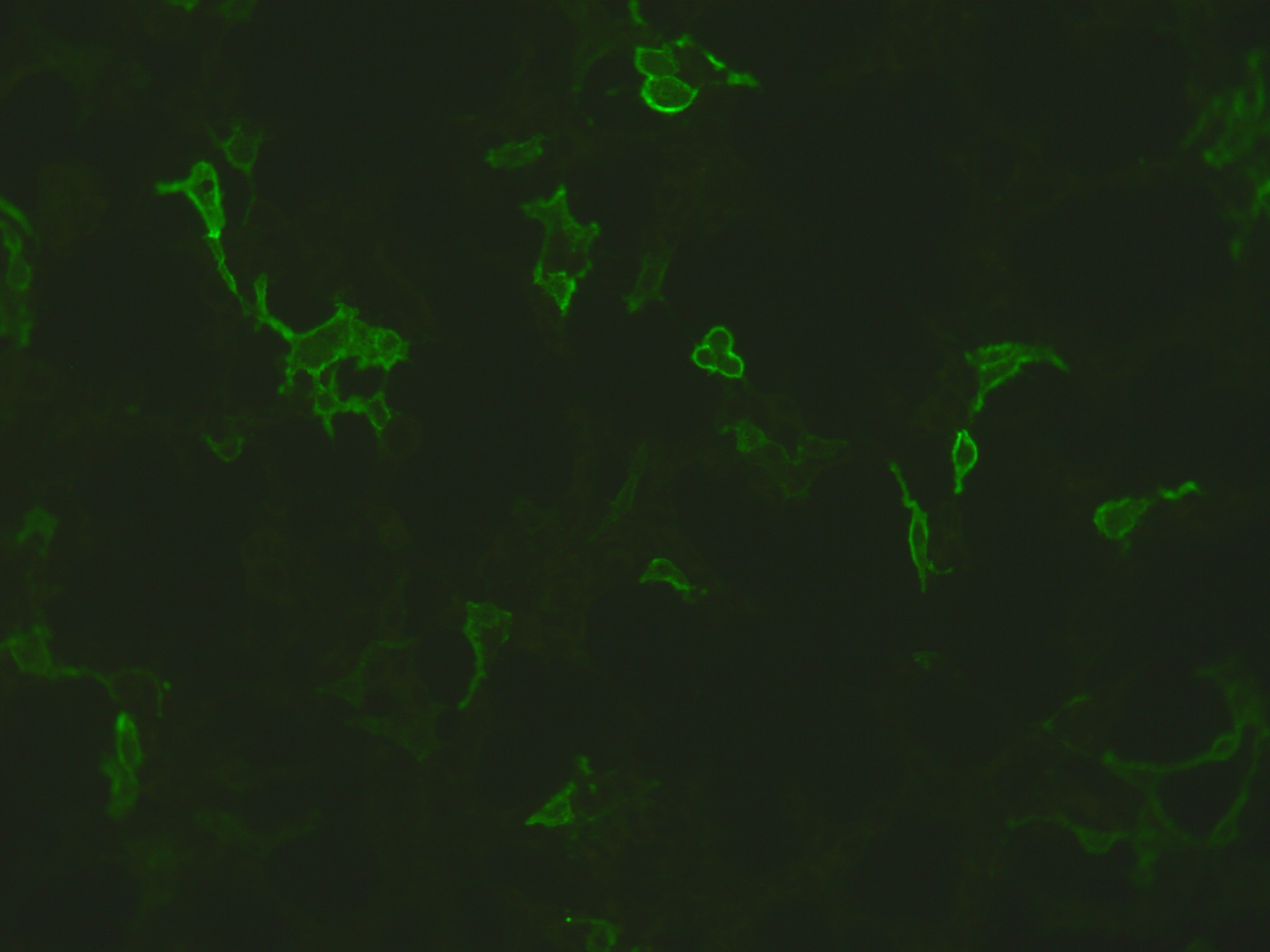


**A**

**B**

**C**
