# Supplementary material for: Clinical features of anti-mGluR5 encephalitis and comparison according to MRI positivity: a systematic review and analysis
Source: Front Immunol. 2026 Jun 5;17:1867988. doi: 10.3389/fimmu.2026.1867988 (PMC13254280; doi:10.3389/fimmu.2026.1867988)
Supplement: Supplementary file 3 [file Table1.pdf]

| Patient no. sex, age (y) | Included in analysis (Yes/No) | Predominant encephalitic phenotype (Yes/No) | Prodromal features | Main clinical features; worst mRS | Tumor | CSF analysis | Brain MRI | Antibody titers | Treatment | Last follow-up, mo; outcome; mRS score | Confounding factors | Study |
|---|---|---|---|---|---|---|---|---|---|---|---|---|
| 1, M, 15 | Yes | Yes | Headache, nausea | Confusion, anxiety, agitation, auditory and visual hallucinations, decreased attention and verbal output, attention deficit, status epilepticus; (5) | HD, stage 2A | 114 WBC, increased IgG index, OCB + | Bilateral hyperintensities in the posterior parietal-occipital cortex | S: +, CSF: NA | Chemotherapy, radiotherapy | 24, complete recovery; (0) | None | Lancaster et al. (2011) |
| 2, F, 46 | Yes | Yes | Personality change | Seizures, personality change, emotional liability, memory impairment, delusions, myoclonus, tremor; (4) | HD, stage 3A | 23WBC | Subtle increase in T2 signal in the right mesial temporal area; 1 mo, increased T2 signal in the mesial temporal lobes, cingulate gyrus, insular regions, and right thalamus | S: +, CSF: NA | Chemotherapy, IVMP | 48; complete recovery; (0) | None | Lancaster et al. (2011) |
| 3, M, 35 | Yes | Yes | Weight loss (9kg) | Memory impairment, personality change, aggressive behavior, right-sided 10th, 11th, and 12th cranial nerve palsies, (MoCA: 16); (4) | HD, stage 2B | 12 WBC, increased IgG index, OCB - | Increased T2 signal and postcontrast enhancement on T1-weighted sequences in the upper pons; post-reatment, normal | S: -, CSF: 1:160 | Chemotherapy, steroids | 38; complete recovery; (0) | None | Mat et al. (2013) |
| 4, F, 30 | Yes | Yes | Weight loss (22 kg), flu-like symptoms | Personality change, aggressive behavior, hypersomnia, memory impairment, visuospatial deficit, prosopagnosia, dLOC, seizures. mRS score 3. Relapse at 16 mo. mRS score 2 | None | 25 WBC, OCB + | Normal | S: 1: 1280, CSF: 1: 320; IgG1, IgG3 | Steroids, PE, RTX | 48; Partial recovery, Mild residual attention deficit; (1) | None | Prüss et al. (2014) |
| 5, M, 68 | Yes | Yes | Fever, weight loss | Inattention, disorientation, confusion, delusions, auditory hallucinations, psychomotor agitatioin, memory impairment, night sweats; (4) | HD, stage 2B | <5WBC, OCB + | Normal | S: NA, CSF: + | IVIg, steroids, chemotherapy | 1; Partial recovery, mild residual memory deficits; (1) | None | Guevara et al. (2018) |
| 6, M, 75 | Yes | Yes | Weight loss (11 kg) | Progressive ophthalmoplegia, postural hand tremor, gait instability, executive dysfunction; (4) | SCLC | 6 WBC, increased IgG index | Bilateral mesiotemporal lobes | S: 1:160, CSF: 1:320 | Chemotherapy, radiotherapy, steroids, IVIg | 62; partial recovery, improved cognition, ophthalmoplegia unchanged; (3) | None | Spatola et al. (2018) |
| 7, F, 6 | Yes | Yes | Rash, headache, flu-like symptoms | Status epilepticus, dLOC, aphasia, memory impairment, poor sleep with altered sleep-wake cycle, followed by dystonia and oculogyric crisis, psychomotor slowness, ataxia, speech and motor regression, hypoventilation; (5) | None | 21 WBC, OCB - | Bilateral frontal and right occipital lobes, cerebellum | S: NA, CSF: 1:10 | Steroids, IVIg, RTX | 19; partial recovery, improved apahsia, can not walk unassistedly; (3) | None | Spatola et al. (2018) |
| 8, M, 15 | Yes | Yes | None | Facial paralysis, then developed altered behavior, memory impairment, anxiety, irritability, visual hallucinations, insomnia; (4) | HD, stage 1 | 45 WBC, OCB + | Normal | S: 1:1280, CSF: 1:640; IgG1, IgG2, IgG3 | Steroids, IVIg, chemotherapy | 12; Moderate memory problems; (2) | None | Spatola et al. (2018) |
| 9, F, 40 | Yes | Yes | Headache | Insomnia, anxiety, psychosis, auditory hallucinations, memory impairment, dLOC, akinetic mutism, orofacial dyskinesia; (5) | None | 45 WBC | Normal | S>> 1:1280, CSF: NA; IgG1 | Steroids, PE | 20; Complete recovery; (0) | None | Spatola et al. (2018) |
| 10, M, 16 | Yes | Yes | Headache | Psychosis, hallucinations, sleep disturbances, dystonia, generalized seizures, dLOC. mRS score 4. After complete recovery, neurologic relapse followed by tumor relapse | HD, stage 3B | 31 WBC, OCB + | Normal | S: >> 1:1280, CSF: 1:20; IgG1, IgG3 | Steroids, PE, chemotherapy | 48; Complete recovery; (0) | None | Spatola et al. (2018) |
| 11, F, 20 | Yes | Yes | Headache, flu-like symptoms | Psychosis, emotional lability, thought disorder, memory impairment, psychomotor slowing, hypersomnia; (4) | None | 27 WBC, OCB + | Normal | S: >> 1:1280, CSF: NA; IgG1, IgG2 | None | 96; Complete recovery; (0) | None | Spatola et al. (2018) |
| 12, M, 49 | Yes | Yes | None | Insomnia, altered behavior, mania, emotional lability, psychomotor agitation, dLOC, seizures; (4) | None | 75 WBC, OCB + | Normal | S: 1:320, CSF: 1:160; IgG1, IgG3 | Steroids | 5; Mild verbal memory and executive deficits; (1) | None | Spatola et al. (2018) |
| 13, M, 38 | No | Yes | Headache, fever | Motor aphasia, psychomotor agitation, tonic-clonic seizures and status epilepticus, (MMSE: 28); (4) | None | Pleocytosis, elevated opening pressure (380mm$H_2O$) | Extensive cortical edema in left cerebral hemisphere | S: 1:10, CSF: 1:10 | IVMP, IVIg, MMF | 6; complete recovery; (0) | MOG: S: 1:32, CSF: 1:10; NMDAR: CSF: 1:10 | Fu et al. (2022) |
| 14, F, 65 | No | Yes | None | Faciobrachial dystonic seizures, dLOC, confusion, positive left Babinski sign; (3) | None | <5 WBC | DWI showed diffusion restriction in the right putamen and caudate nucleus, with corresponding low ADC values | S: 1:10, CSF: - | IVMP, IVIg | 3; complete recovery; (0) | LGI1: S: 1:100, CSF:1:30 | Huo et al. (2022) |
| 15, F, 61 | No | No | None | Focal motor seizures; (2) | None | 8 WBC | T2/FLAIR sequences revealed high signal intensity in the right frontal cortex and subcortical regions; 5 mo, no change | S: +, CSF: - | IVMP | 5; partial recovery, mild residual seizures; (1) | None | Jia et al. (2022) |
| 16, F, 26 | No | Yes | None | Irritability, babbling, stiffness of the limbs, sleepwalking, hallucinations and paroxysmal mania, somnolence, attention deficits; (3) | Bilateral ovarian teratomas (left immature, right mature) | 4 WBC | SCC were characterized by slight hypointensity on T1WI, hyperintensity on T2WI, FLAIR, and DWI, and hypointensity on ADC map (RESLES) | S: 1:10, CSF: - | IVMP, IVIg, chemotherapy, surgery | 3; complete recovery; (0) | NMDAR: S: 1:32, CSF: 1:3.2; 3 mo, S: 1:10 | Li et al. (2022) |

| | | | | | | | | | | | | |
|---|---|---|---|---|---|---|---|---|---|---|---|---|
| 17, M, 44 | No | No | None | Bilateral limb weakness, extremity pain, headache, nausea, red rashes, areflexia, urinary retention, cranial nerve involvement (blurred vision, weakness in chewing, difficulty opening mouth, bitter taste, facial paralysis, dysphagia, choking on water, dysarthria); (3) | None | <5 WBC, albumino-cytological dissociation, OCB + | Normal | S: 1:30, CSF: 1:10; 1mo, S: 1:10, CSF: 1:10 | IVIg | 6; complete recovery; (0) | None | Yan et al. (2022) |
| 18, M, 52 | Yes | Yes | None | Mental abnormality, decreased verbal output, frequent seizures, time and place disorientation; (4) | post-HSE | 19 WBC | FLAIR hyperintensities in the left temporal, occipital, and insula lobes | S: 1:10, CSF: 1:100 | IVMP | 1; partial recovery, mildly recovered from severe mental disorder; (4) | None | Chen et al. (2023) |
| 19, F, 22 | Yes | Yes | None | insomnia and somnolence, memory impairment, positive bilateral Babinski signs, (MoCA: 21); (3) | None | 7 WBC | Patchy FLAIR hyperintensities in the bilateral basal ganglia, insula, and medial temporal lobes | S: 1:32, CSF: 1:10 | IVMP | 5; partial recovery; (1) | None | Chen et al. (2023) |
| 20, M, 51 | Yes | Yes | None | Personality changes, hallucinations, delusions, sleep disturbances, (MoCA: 7); (4) | None | 16 WBC | Abnormal signals in bilateral medial temporal lobes | S: 1:10, CSF: - | IVMP | 4; partial recovery; (2) | None | Chen et al. (2023) |
| 21, M, 58 | Yes | Yes | None | Absence seizures, memory impairment; (2) | None | 1 WBC | Hyperintense FLAIR lesions in the bilateral hippocampus and left insula lobe | S: 1:100, CSF: - | Steroids | 2; partial recovery; (1) | None | Chen et al. (2023) |
| 22, M, 36 | Yes | Yes | None | Generalized tonic-clonic seizures, cognitive deficits, apathy; (3) | None | <5WBC, normal IgG index, OCB + | Normal | S: 1:10, CSF: - | IVIg, steroids | 6; partial recovery, mild residual aphasia; (1) | None | Chen et al. (2023) |
| 23, M, 36 | No | Yes | Headache, flu-like symptoms | Acute onset personality changes, behavioral changes with irritability, mania, visual hallucination, difficulties falling asleep, visual deficits; (2) | None | 80 WBC, increased IgG index, OCB - | T2/FLAIR hyperintensities in unilateral (right) mesiotemporal lobe, cerebral peduncle, thalamus, and putamen | S: -, CSF: 1:10 | IVMP, IVIg | 24; complete recovery; (0) | Recoverin (S) | Guo et al. (2023) |
| 24, F, 35 | No | Yes | Headache | Acute onset spatial disorientation, prosopagnosia, memory deficits, visual hallucination, generalized seizures, refractory status epilepticus, then rapidly progressive dLOC then being in a coma, dystonia, hypoventilation; (5) | Mature teratoma | 120 WBC, increased IgG index | T2/FLAIR hyperintensities in bilateral hippocampi | S: 1:10, CSF: 1:100 | IVMP, IVIg, surgery | 6; death; (6) | NMDAR (CSF and S); AMPAR1 and AMPAR2 (CSF) | Guo et al. (2023) |
| 25, M, 59 | Yes | Yes | Diarrhea, flu-like symptoms | Acute onset personality changes, behavioral changes with irritability, aggressive behavior, visual hallucination, aphasia, memory impairment, dLOC, meningeal irritation, hypoventilation; (5) | None | <5 WBC, normal IgG index, OCB - | Diffuse dura mater enhancement on contrast-enhanced T1WI | S: 1:100, CSF: 1:10 | IVMP | 11; partial recovery, residual cognitive disorders, can not walk unassistedly; (4) | None | Guo et al. (2023) |
| 26, M, 32 | Yes | Yes | Headache, fever | Acute onset personality changes, behavioral changes with irritability, aggressive behavior, apathy, auditory hallucination, memory deficits, attention deficits, difficulties falling asleep; (3). Relapse at 3 month (mRS score 3) and at 28 month (mRS score 3). | None | <5WBC, increased IgG index, OCB NA | Normal at onset and first relapse, T2/ FLAIR hyperintensities in bilateral hippocampi at second relapse | S: 1:320, CSF: 1:10; IgG1, IgG2 (S and CSF) | IVMP, IVIg, followed by tapering oral prednisone; MMF after the second relapse | 42; partial recovery, stable after the second relapse, mild residual memory deficits and attention deficits; (1) | None | Guo et al. (2023) |
| 27, F, 35 | Yes | Yes | Fever,flu-like symptoms | Acute onset personality changes, psychosis, auditory hallucination, decreased verbal output, apathy, depressed mood, memory impairment, executive dysfunction (MoCA: 21), sleep disturbances; (3) | None | <5WBC, normal IgG index, OCB NA. | Normal | S: 1:10, CSF: -; IgG subclass N.A. | IVMP, followed by tapering oral prednisone | 18; complete recovery; (0) | None | Guo et al. (2023) |
| 28, F, 54 | Yes | Yes | None | Verbal memory encoding, recall deficiency, headache; (2) | None | Pleocytosis | Mild cerebral microangiopathy and nonspecific gliosis in the subcortex | S: 1:32, CSF: NA; 3.5 mo, S: - | IVMP, steroids | 3, partial recovery, mild residual cognitive deficits; (1) | None | Hansen et al. (2023) |
| 29, F, 16 | No | Yes | Headache | Only a single generalized tonic-clonic seizure, followed by weight loss, nocturnal awakening, constipation, irritable, crying, anxiety, memory impairment; (2) | None | 3 WBC, normal IgG index, OCB + | Abnormal signals of the R hippocampus | S: +, CSF: - | Steroids, IVIg, MMF | 18; complete recovery; (0) | LGI1 (S: +, CSF: -) | Sun et al. (2023) |
| 30, M, 17 | Yes | Yes | None | Memory impairment, (MoCA: 27), focal seizures with impaired awareness; (2) | None | 1 WBC, normal IgG index, OCB - | T2/FLAIR hyperintensities in Bi medial temporal lobes and insula, enlarged L amygdala | S: +, CSF: - | IVIg | 12; complete recovery; (0) | None | Sun et al. (2023) |
| 31, M, 29 | No | No | None | Focal to bilateral tonic-clonic seizures; (1) | None | 0 WBC | Ischemic foci in white matter, enhancement of the local diploe | S: +, CSF: - | Steroids | 15; complete recovery; (0) | Amphiphysin (S: +, CSF: -) | Sun et al. (2023) |
| 32, F, 70 | Yes | Yes | None | Auditory and visual hallucinations, persecutory delusions, mumble to herself, difficulty in falling and sustaining sleep, nightmares, memory impairment; (3) | None | 1 WBC, normal IgG index, OCB - | Subdural effusion in the R frontotemporal region | S: +, CSF: - | Steroids, IVIg | 12; complete recovery; (0) | None | Sun et al. (2023) |
| 33, F, 46 | Yes | Yes | None | Transient numbness and weakness in left lower limb, then complex partial seizure, memory impairment, (MoCA: 13), depression; (2) | None | 1 WBC, normal IgG index, OCB - | T2/FLAIR hyperintensities in R hippocampal, medial temporal and centrum semiovale white matter, mild cerebral atrophy | S: +, CSF: - | Steroids | 12; complete recovery; (0) | None | Sun et al. (2023) |
| 34, F, 38 | No | No | Fever, diarrhea, flu-like symptoms | Asthenia, anorexia, nausea, vomiting and emotional instability; (1) | None | 9 WBC, normal IgG index, OCB - | Enhancement in R cerebellar tentorium; at 1 year: L pontine demyelination and R frontal white matter lesion; at 2 years: normal | S: +, CSF: - | None | 58; relapse after 2 years (Skin rash, asthenia, anorexia, nausea and vomiting, and emotional instability), then complete recovery; (0) | None | Sun et al. (2023) |

| | | | | | | | | | | | | |
|---|---|---|---|---|---|---|---|---|---|---|---|---|
| 35, F, 78 | No | Yes | Flu-like symptoms | Mild dizziness, walking difficulty due to persist left limb weakness, daytime sleepiness, memory impairment, depression, anxiety and manic, spatiotemporal disorientation, impairment of verbal comprehension, distension, weight loss, incontinence; (5) | None | 13 WBC, normal IgG index, OCB + | Ring enhancement of multiple thin-walled well-circumscribed lesions with varying sizes | S: +, CSF: + | Steroids, IVIg | 15; significant improvement of walking difficulty; (3) | Human herpesvirus (types 6B) in CSF | Sun et al. (2023) |
| 36, M, 49 | No | Yes | None | Dizziness, diplopia, then numbness of both feet, unable to walk, wheelchair bound, status epilepticus, words are difficult to understand, dysphagia, paroxysmal involuntary movements in upper limbs and mandibular. Two febrile events (peak fever temperature 38 °C) during the course of illness; (5) | None | 2 WBC, normal IgG index, OCB - | Subdural effusion in the R frontotemporal region | S: +, CSF: - | IVIg, steroids | NA | Streptococcus mitis in CSF | Sun et al. (2023) |
| 37, M, 65 | Yes | Yes | Fever | Slurred speech, neck stiffness, vomiting, somnolence and mutism, no reaction to pain in the upper limbs, generalized seizures; (5) | None | 29 WBC, increased IgG index, OCB - | Subdural effusion | S: +, CSF: + | None | 12; complete recovery; (0) | None | Sun et al. (2023) |
| 38, M, 71 | Yes | Yes | Heat, pain, redness and swelling in the left ear, headache and fever | Auditory and visual hallucinations, babbling and persecutory delusion, not recognizing friends or family, memory loss, (MoCA: 26), focal seizures with impaired consciousness, slow responses; constipation and slight alterations of continence; (3) | None | 163 WBC, increased IgG index, OCB + | Increased FLAIR signal in hippocampus, medial temporal and subcortical matter of frontal lobes bilaterally | S: +, CSF: - | None | 12; complete recovery; (0) | None | Sun et al. (2023) |
| 39, M, 58 | Yes | Yes | Weight loss | Auditory hallucination, depression, apathy, irritability, REM sleep behavior disorder and reduced sleep duration, dizziness and headache, constipation, urinary retention, memory impairment and spatial disorientation (MOCA: 20); (3) | None | 5 WBC, increased IgG index, OCB - | Normal | S: +, CSF: - | Steroids | 18; complete recovery; (0) | None | Sun et al. (2023) |
| 40, M, 30 | Yes | Yes | Diarrhea | Focal seizures with impaired awareness and focal to bilateral tonic-clonic seizures, emotional lability, memory impairment (MOCA: 29); (1) | None | 3 WBC, normal IgG index, OCB - | Normal | S: +, CSF: - | None | 12; complete recovery; (0) | None | Sun et al. (2023) |
| 41, F, 68 | Yes | Yes | None | Memory impairment, headache with tinnitus, daytime sleepiness, focal seizures with impaired awareness and focal to bilateral tonic-clonic seizures; urinary and fecal incontinence; (5) | None | 2 WBC, | Normal | S: +, CSF: - | Steroids, IVIg | 12; significant improvement; (1) | None | Sun et al. (2023) |
| 42, F, 22 | Yes | Yes | None | Frequent focal seizures without impaired awareness, nocturnal awakening, fever; (2) | None | 2 WBC, normal IgG index, OCB - | Normal | S: +, CSF: + | IVIg, AZA | 15; mild improvement of seizures; (1) | None | Sun et al. (2023) |
| 43, F, 19 | Yes | Yes | None | Focal seizures with impaired awareness, anxiety, memory impairment (MOCA: 29), sleep disturbances; (2) | None | 4 WBC, IgG index NA, OCB - | Normal; at two years follow-up, normal | S: +, CSF: NA | Steroids, PP, MMF | 81; partial recovery; (1) | None | Sun et al. (2023) |
| 44, F, 19 | Yes | Yes | Weight loss | Orthostatic leg tremor, truncal ataxia, sweating, anxiety; (3) | None | <5 WBC, OCB + | Normal | S: 1:1000, CSF: 1:32; 1.5 mo, S: 1:32, CSF: NA | IVIg, IVMP | 18, complete recovery; (0) | None | Yang et al. (2023) |
| 45, M, 29 | Yes | Yes | Headache, flu-like symptoms | Cerebellar ataxia, psychomotor agitation, (MMSE: 28), anxiety, (SAS:65), confusion, brainstem involvement (hiccups, abnormal BAEP), headache, neck rigidity; (4) | None | 297 WBC | Abnormal signals in the splenium of the corpus callosum (RESLES) | S: 1:10, CSF: +; 3 mo, S: 1:10; 6 mo, S: - | IVMP, IVIg | 6; complete recovery; (0) | None | Zhang et al. (2023) |
| 46, F, 7 | Yes | Yes | None | Focal seizures, behavioral disturbance, dystonia, self-talk; (3) | post-HSCT | <5 WBC, OCB - | Normal | S: 1:1000, CSF: NA | IVIg, IVMP | 17; complete recovery; (0) | None | Zhang et al. (2023) |
| 47, F, 60 | No | No | None | Vision loss, headache; (3) | None | 1 WBC | Patchy hyperintensity in the posterior horn of the left ventricle and the left optic nerve on T2WI,T2-FLAIR, DWI; 1 mo, regression of the lesions | S: 1:32, CSF: -; 1 mo, S: 1:10 | IVMP, MMF | 1; partial recovery, mild residual visual impairment; (1) | MOG: S: 1:10, CSF: - | He et al. (2024) |
| 48, F, 30 | Yes | Yes | None | Visual hallucinations, nystagmus, memory impairment, mutism and disorientation, paratonia and abnormal frontal reflexes; (4) | HD | 80 WBC | Involvement of limbic and extra-limbic regions and brainstem | S: +, CSF: + | NA | NA | None | Pedrosa et al. (2024) |
| 49, M, 12 | Yes | Yes | Headache, fever | Auditory hallucination, sleep disturbances, irritability, memory impairment, decreased comprehension; (4) | Gangliocytoma | 90 WBC, OCB + | Speckled abnormality in the right insular lobe | S: 1:100, CSF: 1:3.2 | IVIg, IVMP, surgery | 1; partial recovery, mild residual hallucinations; (1) | None | Shi et al. (2024) |
| 50, M, 69 | Yes | Yes | Weight loss, flu-like symptoms | Myoclonus, truncal ataxia, seizures, psychosis, memory impairment, prosopagonsia, hypoethesia, sleep disturbances, ocular motor dysfunction, visual hallucinations, dysarthria, hypophonia, maculopapular rash; (4) | Acinar Adenocarcinoma Gleason 4 + 3 (grade group III) | 16 WBC | Normal; 3 mo normal | S: 1:10, CSF: - | IVMP, IVig, PE, RTX, chemotherapy | 12; partial recovery, mild residual myoclonus; (2) | None | Pa et al. (2024) |

| | | | | | | | | | | | | |
|---|---|---|---|---|---|---|---|---|---|---|---|---|
| 51, M, 31 | No | Yes | None | Seizures, dLOC | Teratoma | None | calcifications in the bilateral hippocampi but no intracranial lipid droplet signals | S: 1:10 | Steroids, IVIg | 33; complete recovery; (0) | Chemical meningoencephalitis. Seizure attributed to rupture of spinal teratoma | Wang et al. (2024) |
| 52, F, 57 | Yes | Yes | Weight loss (10 kg), anorexia | Ataxia, cognitive deficits, irritability, cognitive deficits (MMSE: 25), (MoCA: 14), bilateral Babinski signs positive; (3) | None | 2 WBC, OCB + | Abnormal enhancement in cerebellar dentate nucleus, cerebral peduncle in the mesencephalon, partially mesial temporal area, thalamus, right basal ganglia and posterior limb of the internal capsule | S: 1:32, CSF: - | IVIg, IVMP | 6; partial recovery; (1) | None | Chen et al. (2025) |
| 53, F, 50 | No | Yes | Headache | Agitation, mutism, hallucinations, dLOC, memory impairment, chorea, myoclonus, constipation, urinary retention; (4) | Papillary thyroid carcinoma (PTC) | 196 WBC, OCB + | Leptomeningeal enhancement, left frontotemporal predominant dural thickening; (Atypical); 5 mo, normal | S: -, CSF: 1:30 | IVMP, RTX, SPA-IA, thyroidectomy | 18; complete recovery; (0) | NMDAR: S: 1:10, CSF: 1:100; GFAP: S: -, CSF: 1:32 | Chen et al. (2025) |
| 54, F, 21 | No | Yes | None | Disorganized speech, tangentiality, and agitation, dLOC, tonic-clonic seizures, fever; (5) | Bilateral ovarian teratoma | 140 WBC | Patchy T2WI/FLAIR hyperintensity and abnormally elevated DWI signals in bilateral hippocampus and right temporoparietal lobe | S: 1:30, CSF: 1:30 | IVMP, IVIg | 2; death; (6) | NMDAR: S: 1:100, CSF: 1:100 | Gu et al. (2025) |
| 55, M, 51 | No | Yes | None | Refractory epilepsy, abnormal mental behaviors, and memory impairment | None | NA | Lesions in the deep right temporal lobe and occipital lobe | S: NA, CSF: + | Steroids | 1; death; (6) | GAD65: S: NA, CSF: +; GABAARα1: S: NA, CSF: + | Peng et al. (2025) |
| 56, M, 39 | Yes | Yes | Persistent fever | Focal to bilateral tonic-clonic seizures, psychosis, incoherent speech, apathy, agitation, drowsiness, memory impairment, disorientation, reduced appetite; (3) | None | Pleocytosis, increased IgG index, OCB pattern 2 | Normal | S: 1:160, CSF: 1:640 | Intravenous methylprednisolone pulses with sustained remission achieved through rituximab therapy combined with a gradual tapering of steroids | 12; complete recovery; (0) | None | Niu et al. (2025) |
| 57, M, 21 | No | No | None | Focal to bilateral tonic-clonic seizures; status epilepticus; (1) | None | NA | Abnormal signals in the bilateral subcortical frontal white matter and periventricular regions | S: 1:32, CSF: 1:1 | IVMP, IVIg | NA | HSV-2 | Liu et al. (2026) |
| 58, M, 50 | No | No | Transient, recurrent episodes of dizziness | Persistent dizziness, hyperreflexia in the lower limbs; (1) | None | <5 WBC | Normal | S: 1:32, CSF: - | Steroids | NA | Hu | Liu et al. (2026) |
| 59, F, 36 | No | Yes | None | Depression, fatigue, insomnia, horizontal nystagmus; (2) | Ovarian mature cystic teratoma. | <5 WBC | Normal | S: 1:10, CSF: -; 8 mo, S:-, CSF: - | IVMP, MMF, RTX | 8; partial recovery, residual emotioinal symptoms; (1) | Ri: S: 1:30, CSF: 1:10; 8 mo, S: -, CSF: - | Wang et al. (2026) |
| 60, F, 18 | Yes | Yes | Weight loss (15 kg) | Cerebellar ataxia, generalized tonic-clonic seizures, syncope, spontaneous pain, hypoesthesia; (4) | None | 3 WBC, OCB pattern 2 | Normal | S: 1:32, CSF: -; 6 mo, S: 1:100, CSF: NA | IVIg, steroids | 12; complete recovery; (0) | None | This study |

Supplementary table 1. All cases of patients with mGluR5 antibody. Abbreviations: Ab = antibody; AZA = azathioprine; Bi = bilateral; FLAIR = fluid-attenuated inversion recovery; IgG = immunoglobulin G; IVIg = intravenous immunoglobulin; L = left; mGluR5 = metabotropic glutamate receptor 5; m = month; MMF = mycophenolate mofetil; RTX = rituximab; mRS = modified Rankin Scale; NA = not available; CSF = cerebrospinal fluid; OCB = oligoclonal bands; PP = plasmapheresis; R = right; WBC = white blood cells per mm³; -/+ = sample negative/positive for mGluR5 cell-based assay.
