# Supplementary material for: Clinical features of anti-mGluR5 encephalitis and comparison according to MRI positivity: a systematic review and analysis
Source: Front Immunol. 2026 Jun 5;17:1867988. doi: 10.3389/fimmu.2026.1867988 (PMC13254280; doi:10.3389/fimmu.2026.1867988)
Supplement: Supplementary file 4 [file Table2.pdf]

Patient no. sex, age (y)	Prodromal features	Main clinical features; worst mRS	Tumor	CSF analysis	Brain MRI	Antibody titers	Treatment	Last follow-up, mo; outcome; mRS score	Study
1, M, 15	Headache, nausea	Confusion, anxiety, agitation, psychosis (auditory and visual hallucinations), decreased attention and verbal output, attention deficit, status epilepticus; (5)	HD, stage 2A	114 WBC, increased IgG index, OCB +	Bilateral hyperintensities in the posterior parietal-occipital cortex	S: +, CSF: NA	Chemotherapy, radiotherapy	24, complete recovery; (0)	Lancaster et al. (2011)
2, F, 46	Personality change	Seizures, personality change, emotional liability, memory impairment, delusions, myoclonus, tremor; (4)	HD, stage 3A	23WBC	Subtle increase in T2 signal in the right mesial temporal area; 1 mo, increased T2 signal in the mesial temporal lobes, cingulate gyrus, insular regions, and right thalamus	S: +, CSF: NA	Chemotherapy, IVMP	48; complete recovery; (0)	Lancaster et al. (2011)
3, M, 35	Weight loss (9kg)	Memory impairment, personality change, aggressive behavior, right-sided 10th, 11th, and 12th cranial nerve palsies, (MoCA: 16); (4)	HD, stage 2B	12 WBC, increased IgG index, OCB -	Increased T2 signal and postcontrast enhancement on T1-weighted sequences in the upper pons; post-reatment, normal	S: -, CSF: 1:160	Chemotherapy, steroids	38; complete recovery; (0)	Mat et al. (2013)
4, F, 30	Weight loss (22 kg), flu-like symptoms	Personality change, aggressive behavior, hypersomnia, memory impairment, visuospatial deficit, prosopagnosia, dLOC, seizures. mRS score 3. Relapse at 16 mo. mRS score 2	None	25 WBC, OCB +	Normal	S: 1: 1280, CSF: 1: 320; IgG1, IgG3	Steroids, PE, RTX	48; Partial recovery, Mild residual attention deficit; (1)	Prüss et al. (2014)
5, M, 68	Fever, weight loss	Inattention, disorientation, confusion, delusions, auditory hallucinations, psychomotor agitatioin, memory impairment, night sweats; (4)	HD, stage 2B	<5WBC, OCB +	Normal	S: NA, CSF: +	IVIg, steroids, chemotherapy	1; Partial recovery, mild residual memory deficits; (1)	Guevara et al. (2018)
6, M, 75	Weight loss (11 kg)	Progressive ophthalmoplegia, postural hand tremor, gait instability, executive dysfunction; (4)	SCLC	6 WBC, increased IgG index	Bilateral mesiotemporal lobes	S: 1:160, CSF: 1:320	Chemotherapy, radiotherapy, steroids, IVIg	62; partial recovery, improved cognition, ophthalmoplegia unchanged; (3)	Spatola et al. (2018)
7, F, 6	Rash, headache, flu-like symptoms	Status epilepticus, dLOC, aphasia, memory impairment, sleep disturbances (poor sleep with altered sleep-wake cycle, followed by dystonia and oculogyric crisis), psychomotor slowness, ataxia, speech and motor regression, hypoventilation; (5)	None	21 WBC, OCB -	Bilateral frontal and right occipital lobes, cerebellum	S: NA, CSF: 1:10	Steroids, IVIg, RTX	19; partial recovery, improved apahsia, can not walk unassistedly; (3)	Spatola et al. (2018)
8, M, 15	None	Facial paralysis, then developed altered behavior, memory impairment, anxiety, irritability, visual hallucinations, insomnia; (4)	HD, stage 1	45 WBC, OCB +	Normal	S: 1:1280, CSF: 1:640; IgG1, IgG2, IgG3	Steroids, IVIg, chemotherapy	12; Moderate memory problems; (2)	Spatola et al. (2018)
9, F, 40	Headache	Insomnia, anxiety, psychosis, auditory hallucinations, memory impairment, dLOC, akinetic mutism, orofacial dyskinesia; (5)	None	45 WBC	Normal	S>> 1:1280, CSF: NA; IgG1	Steroids, PE	20; Complete recovery; (0)	Spatola et al. (2018)
10, M, 16	Headache	Psychosis, hallucinations, sleep disturbances, dystonia, generalized seizures, dLOC. mRS score 4. After complete recovery, neurologic relapse followed by tumor relapse	HD, stage 3B	31 WBC, OCB +	Normal	S: >> 1:1280, CSF: 1:20; IgG1, IgG3	Steroids, PE, chemotherapy	48; Complete recovery; (0)	Spatola et al. (2018)

11, F, 20	Headache, flu-like symptoms	Psychosis, emotional lability, thought disorder, memory impairment, psychomotor slowing, hypersomnia; (4)	None	27 WBC, OCB +	Normal	S: >> 1:1280, CSF: NA; IgG1, IgG2	None	96; Complete recovery; (0)	Spatola et al. (2018)
12, M, 49	None	Insomnia, altered behavior, mania, emotional lability, psychomotor agitation, dLOC, seizures; (4)	None	75 WBC, OCB +	Normal	S: 1:320, CSF: 1:160; IgG1, IgG3	Steroids	5; Mild verbal memory and executive deficits; (1)	Spatola et al. (2018)
13, M, 36	None	Generalized tonic-clonic seizures, cognitive deficits, apathy; (3)	None	<5WBC, normal IgG index, OCB +	Normal	S: 1:10, CSF: -	IVIg, steroids	6; partial recovery, mild residual aphasia; (1)	Chen et al. (2023)
14, M, 52	None	Mental abnormality, decreased verbal output, frequent seizures and cognitive impairment (time and place disorientation); (4)	post-HSE	19 WBC	FLAIR hyperintensities in the left temporal, occipital, and insula lobes	S: 1:10, CSF: 1:100	IVMP	1; partial recovery, mildly recovered from severe mental disorder; (4)	Chen et al. (2023)
15, F, 22	None	Sleep disturbances (insomnia and somnolence), memory impairment, positive bilateral Babinski signs, (MoCA: 21); (3)	None	7 WBC	Patchy FLAIR hyperintensities in the bilateral basal ganglia, insula, and medial temporal lobes	S: 1:32, CSF: 1:10	IVMP	5; partial recovery; (1)	Chen et al. (2023)
16, M, 51	None	Personality changes, hallucinations, delusions, sleep disturbances, (MoCA: 7); (4)	None	16 WBC	Abnormal signals in bilateral medial temporal lobes	S: 1:10, CSF: -	IVMP	4; partial recovery; (2)	Chen et al. (2023)
17, M, 58	None	Absence seizures, memory impairment; (2)	None	1 WBC	Hyperintense FLAIR lesions in the bilateral hippocampus and left insula lobe	S: 1:100, CSF: -	Steroids	2; partial recovery; (1)	Chen et al. (2023)
18, F, 35	Fever,flu-like symptoms	Acute onset personality changes, psychosis, auditory hallucination, decreased verbal output, apathy, depressed mood, memory impairment, executive dysfunction (MoCA: 21), sleep disturbances; (3)	None	<5WBC, normal IgG index, OCB NA.	Normal	S: 1:10, CSF: -; IgG subclass N.A.	IVMP, followed by tapering oral prednisone	18; complete recovery; (0)	Guo et al. (2023)
19, M, 32	Headache, fever	Acute onset personality changes, behavioral changes with irritability, aggressive behavior, apathy, auditory hallucination, memory deficits, attention deficits, difficulties falling asleep; (3). Relapse at 3 month (mRS score 3) and at 28 month (mRS score 3).	None	<5WBC, increased IgG index, OCB NA	Normal at onset and first relapse, T2/ FLAIR hyperintensities in bilateral hippocampi at second relapse	S: 1:320, CSF: 1:10; IgG1, IgG2 (S and CSF)	IVMP, IVIg, followed by tapering oral prednisone; MMF after the second relapse	42; partial recovery, stable after the second relapse, mild residual memory deficits and attention deficits; (1)	Guo et al. (2023)
20, M, 59	Diarrhea, flu-like symptoms	Acute onset personality changes, behavioral changes with irritability, aggressive behavior, visual hallucination, aphasia, memory impairment, dLOC, meningeal irritation, hypoventilation; (5)	None	<5 WBC, normal IgG index, OCB -	Diffuse dura mater enhancement on contrast-enhanced T1WI	S: 1:100, CSF: 1:10	IVMP	11; partial recovery, residual cognitive disorders, can not walk unassistedly; (4)	Guo et al. (2023)
21, F, 54	None	Cognitive deficits (verbal memory encoding and recall deficiency), headache; (2)	None	Pleocytosis	Mild cerebral microangiopathy and nonspecific gliosis in the subcortex	S: 1:32, CSF: NA; 3.5 mo, S: -	IVMP, steroids	3, partial recovery, mild residual cognitive deficits; (1)	Hansen et al. (2023)
22, M, 17	None	Memory impairment, (MoCA: 27), focal seizures with impaired awareness; (2)	None	1 WBC, normal IgG index, OCB -	T2/FLAIR hyperintensities in Bi medial temporal lobes and insula, enlarged L amygdala	S: +, CSF: -	IVIg	12; complete recovery; (0)	Sun et al. (2023)
23, F, 70	None	Auditory and visual hallucinations, persecutory delusions, mumble to herself, sleep disturbances (difficulty in falling and sustaining sleep, nightmares), memory impairment; (3)	None	1 WBC, normal IgG index, OCB -	Subdural effusion in the R frontotemporal region	S: +, CSF: -	Steroids, IVIg	12; complete recovery; (0)	Sun et al. (2023)

24, F, 46	None	Transient numbness and weakness in left lower limb, then complex partial seizure, memory impairment, (MoCA: 13), depression; (2)	None	1 WBC, normal IgG index, OCB -	T2/FLAIR hyperintensities in R hippocampal, medial temporal and centrum semiovale white matter, mild cerebral atrophy	S: +, CSF: -	Steroids	12; complete recovery; (0)	Sun et al. (2023)
25, M, 65	Fever	Slurred speech, neck stiffness, vomiting, somnolence and mutism, no reaction to pain in the upper limbs, generalized seizures; (5)	None	29 WBC, increased IgG index, OCB -	Subdural effusion	S: +, CSF: +	None	12; complete recovery; (0)	Sun et al. (2023)
26, M, 71	Heat, pain, redness and swelling in the left ear, headache and fever	Auditory and visual hallucinations, babbling and persecutory delusion, not recognizing friends or family, memory loss, (MoCA: 26), focal seizures with impaired consciousness, slow responses; autonomic dysfunction (constipation and slight alterations of continence); (3)	None	163 WBC, increased IgG index, OCB +	Increased FLAIR signal in hippocampus, medial temporal and subcortical matter of frontal lobes bilaterally	S: +, CSF: -	None	12; complete recovery; (0)	Sun et al. (2023)
27, M, 58	Weight loss	Auditory hallucination, depression, apathy, irritability, REM sleep behavior disorder and reduced sleep duration, dizziness and headache, constipation, urinary retention, memory impairment and spatial disorientation (MOCA: 20); (3)	None	5 WBC, increased IgG index, OCB -	Normal	S: +, CSF: -	Steroids	18; complete recovery; (0)	Sun et al. (2023)
28, M, 30	Diarrhea	Focal seizures with impaired awareness and focal to bilateral tonic-clonic seizures, emotional lability, memory impairment (MOCA: 29); (1)	None	3 WBC, normal IgG index, OCB -	Normal	S: +, CSF: -	None	12; complete recovery; (0)	Sun et al. (2023)
29, F, 68	None	Memory impairment, headache with tinnitus, daytime sleepiness, focal seizures with impaired awareness and focal to bilateral tonic-clonic seizures; urinary and fecal incontinence; (5)	None	2 WBC,	Normal	S: +, CSF: -	Steroids, IVIg	12; significant improvement; (1)	Sun et al. (2023)
30, F, 22	None	Frequent focal seizures without impaired awareness, nocturnal awakening, fever; (2)	None	2 WBC, normal IgG index, OCB -	Normal	S: +, CSF: +	IVIg, AZA	15; mild improvement of seizures; (1)	Sun et al. (2023)
31, F, 19	None	Focal seizures with impaired awareness, anxiety, memory impairment (MOCA: 29), sleep disturbances; (2)	None	4 WBC, IgG index NA, OCB -	Normal; at two years follow-up, normal	S: +, CSF: NA	Steroids, PP, MMF	81; partial recovery; (1)	Sun et al. (2023)
32, F, 19	Weight loss	Orthostatic leg tremor, truncal ataxia, sweating, anxiety; (3)	None	<5 WBC, OCB +	Normal	S: 1:1000, CSF: 1:32; 1.5 mo, S: 1:32, CSF: NA	IVIg, IVMP	18, complete recovery; (0)	Yang et al. (2023)
33, F, 7	None	Focal seizures, behavioral disturbance, dystonia, self-talk; (3)	post-HSCT	<5 WBC, OCB -	Normal	S: 1:1000, CSF: NA	IVIg, IVMP	17; complete recovery; (0)	Zhang et al. (2023)
34, M, 29	Headache, flu-like symptoms	Cerebellar ataxia, psychomotor agitation, (MMSE: 28), anxiety, (SAS:65), confusion, brainstem involvement (hiccups, abnormal BAEP), headache, meningeal irritation (neck rigidity); (4)	None	297 WBC	Abnormal signals in the splenium of the corpus callosum (RESLES)	S: 1:10, CSF: +; 3 mo, S: 1:10; 6 mo, S: -	IVMP, IVIg	6; complete recovery; (0)	Zhang et al. (2023)

35, M, 69	Weight loss, flu-like symptoms	Myoclonus, truncal ataxia, seizures, psychosis, memory impairment, prosopagonsia, hypoethesia, sleep disturbances, ocular motor dysfunction, visual hallucinations, dysarthria, hypophonia, maculopapular rash; (4)	Acinar Adenocarcinoma Gleason 4 + 3 (grade group III)	16 WBC	Normal; 3 mo follow-up normal	S: 1:10, CSF: -	IVMP, IVig, PE, RTX, chemotherapy	12; partial recovery, mild residual myoclonus; (2)	Pa et al. (2024)
36, F, 30	None	Visual hallucinations, nystagmus, memory impairment, mutism and disorientation, paratonia and abnormal frontal reflexes; (4)	HD	80 WBC	Involvement of limbic and extra-limbic regions and brainstem	S: +, CSF: +	NA	NA	Pedrosa et al. (2024)
37, M, 12	Headache, fever	Auditory hallucination, sleep disturbances, irritability, cognitive deficits (memory impairment, decreased comprehension); (4)	Gangliocytoma	90 WBC, OCB +	Speckled abnormality in the right insular lobe	S: 1:100, CSF: 1:3.2	IVIg, IVMP, surgery	1; partial recovery, mild residual hallucinations; (1)	Shi et al. (2024)
38, M, 39	Persistent fever	Focal to bilateral tonic-clonic seizures, psychosis, incoherent speech, apathy, agitation, drowsiness, memory impairment, disorientation, reduced appetite; (3)	None	Pleocytosis, increased IgG index, OCB pattern 2	Normal	S: 1:160, CSF: 1:640	Intravenous methylprednisolone pulses with sustained remission achieved through RTX therapy combined with a gradual tapering of steroids	12; complete recovery; (0)	Niu et al. (2025)
39, F, 57	Weight loss (10 kg), anorexia	Ataxia, cognitive deficits, irritability, cognitive deficits (MMSE: 25), (MoCA: 14), bilateral Babinski signs positive; (3)	None	2 WBC, OCB +	Abnormal enhancement in cerebellar dentate nucleus, cerebral peduncle in the mesencephalon, partially mesial temporal area, thalamus, right basal ganglia and posterior limb of the internal capsule	S: 1:32, CSF: -	IVIg, IVMP	6; partial recovery; (1)	Chen et al. (2025)
40, F, 18	Weight loss (15 kg)	Cerebellar ataxia, generalized tonic-clonic seizures, dLOC, spontaneous pain, hypoesthesia; (4)	None	3 WBC, OCB pattern 2	Normal	S: 1:32, CSF: -; 6 mo, S: 1:100, CSF: NA	IVIg, steroids	12; complete recovery; (0)	This study

Supplementary table 2. All cases of autoimmune encephalitis patients with mGluR5. Abbreviations: Ab = antibody; AZA = azathioprine; Bi = bilateral; FLAIR = fluid-attenuated inversion recovery; IgG = immunoglobulin G; IVIg = intravenous immunoglobulin; L = left; mGluR5 = metabotropic glutamate receptor 5; m = month; MMF = mycophenolate mofetil; RTX = rituximab; mRS = modified Rankin Scale; NA = not available; CSF = cerebrospinal fluid; OCB = oligoclonal bands; PP = plasmapheresis; R = right; WBC = white blood cells per mm³; -/+ = sample negative/positive for mGluR5 cell-based assay; mo = month.
