# Supplementary material for: Clinical features of anti-mGluR5 encephalitis and comparison according to MRI positivity: a systematic review and analysis
Source: Front Immunol. 2026 Jun 5;17:1867988. doi: 10.3389/fimmu.2026.1867988 (PMC13254280; doi:10.3389/fimmu.2026.1867988)
Supplement: Supplementary file 5 [file Table3.pdf]

Patient no. sex, age (y)	Predominant encephalitic phenotype (Yes/No)	Prodromal features	Main clinical features; worst mRS	Tumor	CSF analysis	Brain MRI	Antibody titers	Treatment	Last follow-up, mo; outcome; mRS score	Confounding factors	Study
1, M, 38	Yes	Headache, fever	Motor aphasia, psychomotor agitation, tonic-clonic seizures and status epilepticus, (MMSE: 28); (4)	None	Pleocytosis, elevated opening pressure (380mmH_2O)	Extensive cortical edema in left cerebral hemisphere	S: 1:10, CSF: 1:10	IVMP, IVIg, MMF	6; complete recovery; (0)	MOG: S: 1:32, CSF: 1:10; NMDAR: CSF: 1:10	Fu et al. (2022)
2, F, 65	Yes	None	Faciobrachial dystonic seizures, dLOC, confusion, positive left Babinski sign; (3)	None	<5 WBC	DWI showed diffusion restriction in the right putamen and caudate nucleus, with corresponding low ADC values	S: 1:10, CSF: -	IVMP, IVIg	3; complete recovery; (0)	LGI1: S: 1:100, CSF:1:30	Huo et al. (2022)
3, F, 61	No	None	Focal motor seizures; (2)	None	8 WBC	T2/FLAIR sequences revealed high signal intensity in the right frontal cortex and subcortical regions; 5 mo, no change	S: +, CSF: -	IVMP	5; partial recovery, mild residual seizures; (1)	None	Jia et al. (2022)
4, F, 26	Yes	None	Irritability, babbling, stiffness of the limbs, sleepwalking, hallucinations and paroxysmal mania, somnolence, attention deficits; (3)	Bilateral ovarian teratomas (left immature, right mature)	4 WBC	SCC were characterized by slight hypointensity on T1WI, hyperintensity on T2WI, FLAIR, and DWI, and hypointensity on ADC map (RESLES)	S: 1:10, CSF: -	IVMP, IVIg, chemotherapy, surgery	3; complete recovery; (0)	NMDAR: S: 1:32, CSF: 1:3.2; 3 mo, S: 1:10	Li et al. (2022)
5, M, 44	No	None	Bilateral limb weakness, extremity pain, headache, nausea, red rashes, areflexia, urinary retention, cranial nerve involvement (blurred vision, weakness in chewing, difficulty opening mouth, bitter taste, facial paralysis, dysphagia, choking on water, dysarthria); (3)	None	<5 WBC, albumino-cytological dissociation, OCB +	Normal	S: 1:30, CSF: 1:10; 1mo, S: 1:10, CSF: 1:10	IVIg	6; complete recovery; (0)	None	Yan et al. (2022)
6, M, 36	Yes	Headache, flu-like symptoms	Acute onset personality changes, behavioral changes with irritability, mania, visual hallucination, difficulties falling asleep, visual deficits; (2)	None	80 WBC, increased IgG index, OCB -	T2/FLAIR hyperintensities in unilateral (right) mesiotemporal lobe, cerebral peduncle, thalamus, and putamen	S: -, CSF: 1:10	IVMP, IVIg	24; complete recovery; (0)	Recoverin (S)	Guo et al. (2023)
7, F, 35	Yes	Headache	Acute onset spatial disorientation, prosopagnosia, memory deficits, visual hallucination, generalized seizures, refractory status epilepticus, then rapidly progressive dLOC then being in a coma, dystonia, hypoventilation; (5)	Mature teratoma	120 WBC, increased IgG index	T2/FLAIR hyperintensities in bilateral hippocampi	S: 1:10, CSF: 1:100	IVMP, IVIg, surgery	6; death; (6)	NMDAR (CSF and S); AMPAR1 and AMPAR2 (CSF)	Guo et al. (2023)
8, F, 16	Yes	Headache	Only a single generalized tonic-clonic seizure, followed by weight loss, nocturnal awakening, constipation, irritable, crying, anxiety, memory impairment; (2)	None	3 WBC, normal IgG index, OCB +	Abnormal signals of the R hippocampus	S: +, CSF: -	Steroids, IVIg, MMF	18; complete recovery; (0)	LGI1 (S: +, CSF: -)	Sun et al. (2023)
9, M, 29	No	None	Focal to bilateral tonic-clonic seizures; (1)	None	0 WBC	Ischemic foci in white matter, enhancement of the local diploe	S: +, CSF: -	Steroids	15; complete recovery; (0)	Amphiphysin (S: +, CSF: -)	Sun et al. (2023)
10, F, 38	No	Fever, diarrhea, flu-like symptoms	Asthenia, anorexia, nausea, vomiting and emotional instability; (1)	None	9 WBC, normal IgG index, OCB -	Enhancement in R cerebellar tentorium; at 1 year: L pontine demyelination and R frontal white matter lesion; at 2 years: normal	S: +, CSF: -	None	58; relapse after 2 years (Skin rash, asthenia, anorexia, nausea and vomiting, and emotional instability), then complete recovery; (0)	None	Sun et al. (2023)
11, F, 78	Yes	Flu-like symptoms	Mild dizziness, walking difficulty due to persist left limb weakness, daytime sleepiness, memory impairment, depression, anxiety and manic, spatiotemporal disorientation, impairment of verbal comprehension, distension, weight loss, incontinence; (5)	None	13 WBC, normal IgG index, OCB +	Ring enhancement of multiple thin-walled well-circumscribed lesions with varying sizes	S: +, CSF: +	Steroids, IVIg	15; significant improvement of walking difficulty; (3)	Human herpesvirus (types 6B) in CSF	Sun et al. (2023)
12, M, 49	Yes	None	Dizziness, diplopia, then numbness of both feet, unable to walk, wheelchair bound, status epilepticus, words are difficult to understand, dysphagia, paroxysmal involuntary movements in upper limbs and mandibular. Two febrile events (peak fever temperature 38 °C) during the course of illness; (5)	None	2 WBC, normal IgG index, OCB -	Subdural effusion in the R frontotemporal region	S: +, CSF: -	IVIg, steroids	NA	Streptococcus mitis in CSF	Sun et al. (2023)
13, F, 60	No	None	Vision loss, headache; (3)	None	1 WBC	Patchy hyperintensity in the posterior horn of the left ventricle and the left optic nerve on T2WI,T2-FLAIR, DWI; 1 mo, regression of the lesions	S: 1:32, CSF: -; 1 mo, S: 1:10	IVMP, MMF	1; partial recovery, mild residual visual impairment; (1)	MOG: S: 1:10, CSF: -	He et al. (2024)

14, M, 31	Yes	None	Seizures, dLOC	Teratoma	None	calcifications in the bilateral hippocampi but no intracranial lipid droplet signals	S: 1:10	Steroids, IVIg	33; complete recovery; (0)	Chemical meningoencephalitis. Seizure attributed to rupture of spinal teratoma	Wang et al. (2024)
15, F, 50	Yes	Headache	Agitation, mutism, hallucinations, dLOC, memory impairment, chorea, myoclonus, constipation, urinary retention; (4)	Papillary thyroid carcinoma (PTC)	196 WBC, OCB +	Leptomeningeal enhancement, left frontotemporal predominant dural thickening; (Atypical); 5 mo, normal	S: -, CSF: 1:30	IVMP, RTX, SPA-IA, thyroidectomy	18; complete recovery; (0)	NMDAR: S: 1:10, CSF: 1:100; GFAP: S: -, CSF: 1:32	Chen et al. (2025)
16, F, 21	Yes	None	Disorganized speech, tangentiality, and agitation, dLOC, tonic-clonic seizures, fever; (5)	Bilateral ovarian teratoma	140 WBC	Patchy T2WI/FLAIR hyperintensity and abnormally elevated DWI signals in bilateral hippocampus and right temporoparietal lobe	S: 1:30, CSF: 1:30	IVMP, IVIg	2; death; (6)	NMDAR: S: 1:100, CSF: 1:100	Gu et al. (2025)
17, M, 51	Yes	None	Refractory epilepsy, abnormal mental behaviors, and memory impairment	None	NA	Lesions in the deep right temporal lobe and occipital lobe	S: NA, CSF: +	Steroids	1; death; (6)	GAD65: S: NA, CSF: +; GABAARα1: S: NA. CSF: +	Peng et al. (2025)
18, M, 21	No	None	Focal to bilateral tonic-clonic seizures; status epilepticus; (1)	None	NA	Abnormal signals in the bilateral subcortical frontal white matter and periventricular regions	S: 1:32, CSF: 1:1	IVMP, IVIg	NA	HSV-2	Liu et al. (2026)
19, M, 50	No	Transient, recurrent episodes of dizziness	Persistent dizziness, hyperreflexia in the lower limbs; (1)	None	<5 WBC	Normal	S: 1:32, CSF: -	Steroids	NA	Hu	Liu et al. (2026)
20, F, 36	Yes	None	Depression, fatigue, insomnia, horizontal nystagmus; (2)	Ovarian mature cystic teratoma.	<5 WBC	Normal	S: 1:10, CSF: -; 8 mo, S:-, CSF: -	IVMP, MMF, RTX	8; partial recovery, residual emotioinal symptoms; (1)	Ri: S: 1:30, CSF: 1:10; 8 mo, S: -, CSF: -	Wang et al. (2026)

Supplementary table 3. All cases of patients with mGluR5 antibody. Abbreviations: Ab = antibody; AZA = azathioprine; Bi = bilateral; FLAIR = fluid-attenuated inversion recovery; IgG = immunoglobulin G; IVIg = intravenous immunoglobulin; L = left; mGluR5 = metabotropic glutamate receptor 5; m = month; MMF = mycophenolate mofetil; RTX = rituximab; mRS = modified Rankin Scale; NA = not available; CSF = cerebrospinal fluid; OCB = oligoclonal bands; PP = plasmapheresis; R = right; WBC = white blood cells per mm³; -/+ = sample negative/positive for mGluR5 cell-based assay.
