# Supplementary material for: Clinical features of anti-mGluR5 encephalitis and comparison according to MRI positivity: a systematic review and analysis
Source: Front Immunol. 2026 Jun 5;17:1867988. doi: 10.3389/fimmu.2026.1867988 (PMC13254280; doi:10.3389/fimmu.2026.1867988)
Supplement: Supplementary file 6 [file Table4.pdf]

**MRI-positive lesion category**	**n (%) among MRI-positive cases**
Limbic involvement	11/21 (52.4%)
Insular involvement	6/21 (28.6%)
Extra-limbic cortical/subcortical involvement	6/21 (28.6%)
Deep gray matter involvement	3/21 (14.3%)
Brainstem/cerebelllar involvement	4/21 (19.0%)
Atypical or non-specific findings	5/21 (23.8%)

Supplementary table 4. Positive MRI distributions. Categories were not mutually exclusive; one patient could contribute to more than one lesion category.
