# Supplementary material for: Clinical features of anti-mGluR5 encephalitis and comparison according to MRI positivity: a systematic review and analysis
Source: Front Immunol. 2026 Jun 5;17:1867988. doi: 10.3389/fimmu.2026.1867988 (PMC13254280; doi:10.3389/fimmu.2026.1867988)
Supplement: Supplementary file 7 [file Table5.pdf]

	MRI-negative	MRI-positive	Effect size (95%CI)	P-value
Clinical features				
Behavioral and mood disturbances	15/19 (78.9%)	12/17 (70.6%)	+8.4% (-20.0 to +36.7)	0.706
Seizures	11/19 (57.9%)	7/17 (41.2%)	+16.7% (-15.5 to 49.0)	0.505
Cognitive deficits	13/19 (68.4%)	17/17 (100.0%)	-31.6% (-52.5 to -10.1)	0.020
Sleep disturbances	12/19 (63.2%)	5/17 (29.4%)	+33.7% (+3.1 to 64.4)	0.054
Decreased level of consciousness	5/19 (26.3%)	1/17 (5.9%)	+20.4% (-2.3 to +43.2)	0.182
Movement disorders	6/19 (31.6%)	6/17 (35.3%)	-3.7% (-34.6 to +27.2)	1.000
Prodromal symptoms	13/19 (68.4%)	9/17 (52.9%)	+15.5% (-16.1 to 47.1)	0.495
Tumor association	3/19 (15.8%)	6/17 (35.3%)	-19.5% (-47.5 to +8.5)	0.255
CSF analyses				
CSF pleocytosis	8/19 (42.1%)	12/17 (70.6%)	-28.5% (-59.5 to +2.5)	0.106
CSF OCBs	10/15 (66.7%)	4/8 (50.0%)	+16.7% (-25.4 to +58.7)	0.657
CSF antibody +	7/14 (50.0%)	8/14 (57.1%)	+7.1% (-29.7 to +44.0)	1.000
Median mRS (IQR)				
Peak of the disease	3 (3,4)	4 (3,4)	0.0 (-1.0 to +1.0)	0.639
Last follow-up	0 (0,1)	1 (0,1)	0.0 (-1.0 to +1.0)	0.368

Supplementary table 5. Sensitivity analysis. Effect size is presented as Hodges-Lehmann median difference for mRS and risk difference (RD) for other clinical features. Abbreviations: CSF, cerebrospinal fluid; OCB, oligoclonal bands; IQR, interquartile range; CI, confidence interval; +, positivity.
